# Machine learning-based mortality prediction models for smoker COVID-19 patients

**DOI:** 10.1186/s12911-023-02237-w

**Published:** 2023-07-21

**Authors:** Ali Sharifi-Kia, Azin Nahvijou, Abbas Sheikhtaheri

**Affiliations:** 1 Department of Health Information Management, School of Health Management and Information Sciences, Iran University of Medical Sciences, Tehran, Iran; 2 Cancer Research Center, Cancer Institute of Iran, Tehran University of Medical Sciences, Tehran, Iran

**Keywords:** Prediction model, Coronavirus Disease 2019, Mortality, Smoking, Machine learning, Data mining

## Abstract

**Background:**

The large number of SARS-Cov-2 cases during the COVID-19 global pandemic has burdened healthcare systems and created a shortage of resources and services. In recent years, mortality prediction models have shown a potential in alleviating this issue; however, these models are susceptible to biases in specific subpopulations with different risks of mortality, such as patients with prior history of smoking. The current study aims to develop a machine learning-based mortality prediction model for COVID-19 patients that have a history of smoking in the Iranian population.

**Methods:**

A retrospective study was conducted across six medical centers between 18 and 2020 and 15 March 2022, comprised of 678 CT scans and laboratory-confirmed COVID-19 patients that had a history of smoking. Multiple machine learning models were developed using 10-fold cross-validation. The target variable was in-hospital mortality and input features included patient demographics, levels of care, vital signs, medications, and comorbidities. Two sets of models were developed for at-admission and post-admission predictions. Subsequently, the top five prediction models were selected from at-admission models and post-admission models and their probabilities were calibrated.

**Results:**

The in-hospital mortality rate for smoker COVID-19 patients was 20.1%. For “at admission” models, the best-calibrated model was XGBoost which yielded an accuracy of 87.5% and F_1_ score of 86.2%. For the “post-admission” models, XGBoost also outperformed the rest with an accuracy of 90.5% and F_1_ score of 89.9%. Active smoking was among the most important features in patients’ mortality prediction.

**Conclusion:**

Our machine learning-based mortality prediction models have the potential to be adapted for improving the management of smoker COVID-19 patients and predicting patients’ chance of survival.

**Supplementary Information:**

The online version contains supplementary material available at 10.1186/s12911-023-02237-w.

## Background

Complications associated with coronavirus disease (COVID-19) are a major global health concern [1]. COVID-19 leads to upper respiratory infections, resulting in acute respiratory syndrome, pneumonia, cardiac, liver, and kidney injuries, secondary infections, sepsis, and even death with a mortality rate of 2–3% [2–4]. Common symptoms include fever, dry cough, myalgia, anorexia, diarrhea, nausea/vomiting, and anosmia [5–7]. As of February 2023, there has been more than 757 million cases of infection and 6.8 million cases of death worldwide [8]. Reports demonstrated higher mortality and disease severity among active or former tobacco smokers compared to non-smokers [9–12], due to higher likelihood of developing respiratory diseases in smoker populations [13].

A large number of hospitalizations associated with COVID-19 have put an unexpected burden on healthcare systems and resource shortages [14, 15]. Timely and effective healthcare service delivery is an important factor in COVID-19 management [16]. In this regard, machine learning (ML) models have shown great promise for predicting disease prognosis, complication prediction, and, improved patient management [17–19].

ML algorithms have been explored in many aspects of COVID-19 management such as detecting epidemiological outbreaks, identification, and diagnosis of COVID-19, and severity or mortality prediction [20–24]. These ML models are beneficial tools for the management of COVID-19 patients [20, 25–27].

Iran was among the first countries facing widespread COVID-19 and had one of the highest mortality rates [28]. The higher prevalence of infections and scarce healthcare resources warrants a further need for an effective predictive model trained on data from the patients, considering the features of the Iranian population [29]. Furthermore, previous mortality prediction models which were developed during the early period of the pandemic showed low prediction performance and recent models usually suffer from selection bias and training using unbalanced data, which could attribute the high performance of these models in accurately identifying negative cases and excluding positive cases [30, 31]. Additionally, ML models may have a bias in subpopulations with different rates of mortality [20] such as smokers.

To our knowledge, designing a mortality prediction model for COVID-19 patients with a focus on smoking patients has been scarcely investigated. The current study aims to develop ML models for mortality prediction in COVID-19 patients with a history of smoking in the Iranian population. Models in this study were developed for use at the time of admission (at admission) and after patient admission during hospitalization (post-admission).

## Methods

### Data Source and Study Population

Retrospective cohort data were extracted from the Imam Khomeini hospital complex COVID-19 registry, which collects data from hospitalized patients from six hospitals in Tehran. The data is collected when patients are hospitalized and when a change in the level of care occurred (for example admission to the ICU). Eight trained nurses and health information technology specialists collect data from patients’ medical records using a documented protocol and enter the data into the registry software. The cohort included active/former smoker patients with a COVID-19 diagnosis who were admitted to one of six hospitals between 18 and 2020 and 15 March 2022. Patients were included based on positive diagnoses with reverse transcriptase-PCR test or CT scan results.

Features were excluded that based on past evidence were irrelevant to COVID-19 mortality, features that had more than a 30% missing rate, and features that had more than 95% of data distributed in one class. Finally, a dataset comprised of 678 smoker patients with 183 features were extracted and after applying inclusion and exclusion criteria, a total of 678 patients with 31 features were finally analyzed. Table S1 (Additional file 1) lists the 183 variables included in the dataset collected from the registry.

### Data preprocessing

A data point was considered as an outlier if the data had equal to or more than ± 3 standard deviation from the mean of the feature. The outliers were replaced with the upper and lower boundary of the interquartile range.

The numerical values were scaled using normalization and the categorical values were encoded (1 and 0 for “Yes” and “No” values, respectively).

The missingness of 11 variables ranged between 0.15% and 27.64%. For numerical variables that had a skewed distribution, missing values were imputed with the median, and the rest were imputed with the mean. Categorical values were imputed using the highest frequency value. Table S2 (Additional file 1) presents the missing rate of features.

### Features and feature selection

The main outcome is confirmed COVID-19-related in-hospital mortality which was collected as binary (yes/no). The dataset consists of 31 variables including patient demographics (e.g., age, sex, and BMI), sign and symptoms, comorbidities, medication history and medication prescribed in hospitals, and lifestyle factors (e.g. tobacco/narcotic consumption).

Eight different feature sets were developed based on 3 main approaches:

1. Univariate analysis using Chi-square tests for categorical variables and T-test for numerical variables (Feature set 1). Features with p-value less than 0.2 were selected.
2. Applying feature importance algorithms such as recursive feature elimination with cross-validation (RFECV) and Gini importance criteria (Feature set 2–7):
  Feature vectors were used as inputs for RFECV with logistic regression, random forest, and gradient boosting and the top 20 Gini importance criteria for extratreesclassifier, random forest, and gradient boosting were selected. Figures S1-S6 (Additional file 2) show the results of selected features based on Gini importance for “at admission” and “post-admission” models.
3. Physician opinion (Feature set 8):
  We developed and distributed a questionnaire among 32 specialists (including infectious disease specialists, pulmonologists, intensive care specialists, and anesthesiologists) who were asked to identify the mortality risk factors. The Kuder Richardson 20 test was used for testing the reliability of questionnaires (reliability = 0.96). Specialists were asked to identify a factor as important or not important (Yes/No). Factors with more than 60% of the specialists’ agreement were included in this feature set.

### Data balancing

Initially, the base models were developed using XGBoost on different feature sets. As a result, the poor performance of these models due to the imbalanced number of deaths (79.9% surviving vs. 20.1% death cases, ratio of 3.98) was discovered. Table S3 (Additional file 1) shows model performance before balancing the minority class. As a solution, we oversampled the minor class using the synthetic minority oversampling technique (SMOTE) and found an improvement compared to the base models. SMOTE is an oversampling technique which the minority class is synthetically oversampled by selecting examples that are close in the feature space, drawing a line between the examples in the feature space and drawing a new sample at a point along that line [32]. This method has been used for application of machine learning methods for mortality prediction [33]. Subsequently, all models were developed using balanced datasets.

### Model Development, evaluation, and explainability

**Fig. 1:**
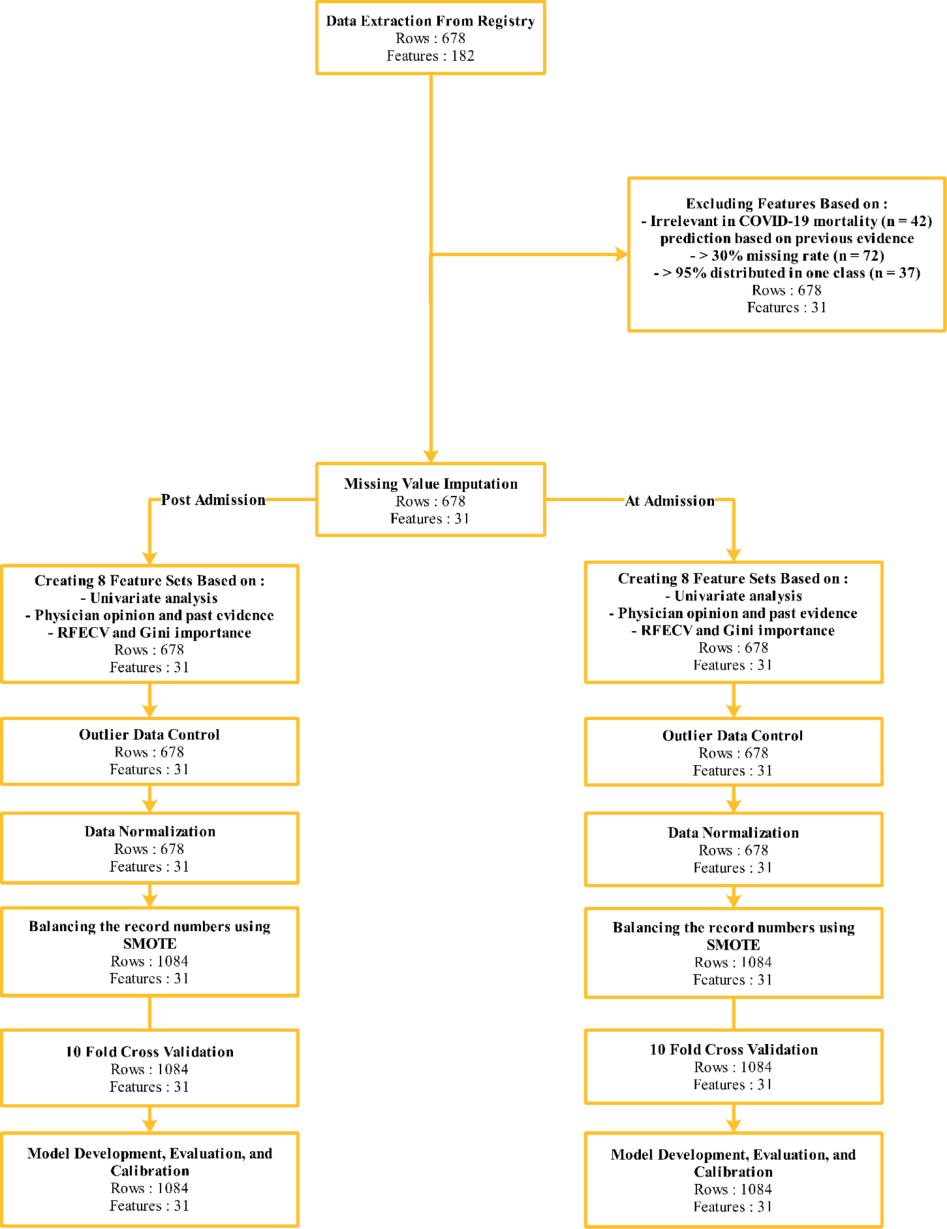
Study Process

Figure 1 depicts the study process. Our binary classification models were developed with eight feature sets utilizing XGBoost, support vector machine (SVM), multi-layer perceptron (MLP), k-nearest neighbor (KNN), random forest (RF), decision tree, logistic regression, and naive Bayes with 10-fold cross-validation.

Logistic regression is a statistical method that uses the sigmoid function as its core method and is used for building machine learning models where the target variable is binary (e.g. death/alive) [34–36]. This algorithm is easy to implement, interpret and train, however, it overfits on high dimensional data and fails to capture complex relationships [37].

Naive Bayes is a binary and multi-class classification algorithm based on the Bayes theorem [38, 39]. This algorithm is a statistical classifier that predicts the probability of membership of a given sample in a specific class. It has a high speed and robust performance on large databases [40, 41].

Furthermore, SVM is a supervised machine learning algorithm used both for classification and regression. SVM will try to find a hyperplane in an n-dimensional space that distinctly classifies the data points [42, 43]. SVM can deal with complex non-linear data points such as health data and is less prone to overfitting [44]. In addition to linear kernel function, SVM can be used as a non-linear kernel function. The most general kernels used in SVM are linear, polynomial, and radial basis function (RBF) [44, 45].

MLP is a type of feed-forward neural network algorithm that consists of interconnected neurons transferring information to each other [46, 47]. To each of the connections between the neurons, a weight has been assigned during training; the weights will be adjusted to learn how to predict the output [44]. MLP is simple and works well with both small and large datasets, however, its computations are complex and time-consuming [48].

Decision tree is a supervised machine learning algorithm used for classification and regression. It has a hierarchical, tree structure which consists of a root node, branches, internal nodes, and leaf nodes [49, 50]. The purpose of this algorithm is to display the structural information stored in the data. This algorithm is fast, easy to use and can handle high dimensional data [44].

Random forest is an ensemble learning algorithm that operates by constructing multiple decision trees and the output is decided by voting [51, 52]. This combined output makes the random forest less prone to noise and outliers compared to a single decision tree [53]. However, computation is very complex and the result could change with a small change occurring in the data [53, 54].

K-nearest neighbor is also a supervised machine learning algorithm used both for classification and regression. This algorithm uses proximity to make classifications or predictions about the grouping of an individual data point [55, 56]. This algorithm is fast and easy to use and understand, however, it has a high computational cost, and it is sensitive to structure of data and requires a large storage space [44].

XGBoost stands for extreme gradient boosting algorithm which is a type of ensemble learning algorithm. It is designed for speed, ease of use, and performance on large datasets [57, 58]. In XGBoost, decision trees are created sequentially and a weight is assigned to all the independent variables which then are given as input to a decision tree. Based on the prediction result, the weights will be adjusted and given as input to another decision tree. This ensemble prediction method will result in more precise and robust model [59].

Furthermore, ensemble models were also developed using aforementioned algorithms on each feature set using Scikit learn ML library and Python (version 3.9.7). Hyperparameters were optimized by creating a parameter list based on each algorithm and using GridSearchCV for identifying the best parameters for each model.

Models were evaluated and compared based on accuracy, the area under the receiver operating characteristics curve (AUC ROC), precision, recall, F_1_ score, logistic loss, and brier score. To select the best-performing model, models were compared based on their F_1_ score and AUC. Afterwards, the top five models were selected from the at-admission and post-admission models and their probabilities were calibrated.

Finally, Shapely additive explanation (SHAP) was applied to provide explainability of the models. SHAP is an approach that is based on cooperative game theory which explains the output of ML models by calculating the contribution of each feature to the prediction [60].

## Results

### Descriptive data

In total, 542 (79.9%) patients survived until discharge from hospitals, and 136 (20.1%) patients expired. Age, oxygen saturation percent (SpO2%), duration of intubation, sweating, abnormal lung signs, hypertension, cancers, cardiovascular diseases, CKD, anti-hypertensive drugs, using pantoprazole, hospitalization 14 days before current admission and admission in an intensive care unit (ICU) were significant factors contributing to patients’ death. Table 1 depicts the basic characteristics of patients.

**Table 1:** Characteristics of surviving vs. non-surviving patients

Variables	Alive (n = 542)	Dead (n = 136)	Total (n = 687)	p-value
Demographic Data				
Age (Year)				< 0.0001
Mean ± SD	56.229 ± 15.38	65.81 ± 14.44	58.15 ± 15.66	
Median	59	69	61	
BMI				0.088
Mean ± SD	26.28 ± 3.69	25.60 ± 3.89	26.14 ± 3.74	
Median	26.1	26.1	26.1	
Average Daily Used Cigarettes (Loosie) (n = 81)	n = 62	n = 19	n = 81	0.295
Mean ± SD	13.50 ± 11.38	15.73 ± 14.26	14.02 ± 12.06	
Median	10	12	10	
Sex				0.082
Female	69 (12.7%)	10 (7.4%)	79 (11.7%)	
Male	473 (87.3%)	126 (92.6%)	599 (88.3%)	
Current Smoking				0.168
No	116 (21.4%)	38 (27.9%)	154 (22.7%)	
Yes	426 (78.6%)	98 (72.1%)	524 (77.3%)	
History of Hookah Consumption				0.334
No	481 (88.7%)	125 (91.9%)	606 (89.4%)	
Yes	61 (11.3%)	11 (8.1%)	72 (10.6%)	
Drug History				0.254
No	373 (68.8%)	86 (63.2%)	459 (67.7%)	
Yes	169 (31.2%)	50 (36.8%)	219 (32.3%)	
Vital Signs				
Systolic Blood Pressure				0.727
Mean ± SD	122.68 ± 19.62	121.37 ± 22.59	122.42 ± 20.24	
Median	122	120	121	
Diastolic Blood Pressure				0.585
Mean ± SD	77.06 ± 12 0.36	74.83 ± 13.29	76.61 ± 12.57	
Median	78	76.50	78	
Respiratory Rate				0.243
Mean ± SD	19.55 ± 5.79	20.36 ± 8.94	19.71 ± 6.54	
Median	19.71	19.71	19.71	
Oxygen Saturation Percent				< 0.0001
Mean ± SD	89.48 ± 7.84	85.40 ± 10.71	88.66 ± 8.64	
Median	91	88.50	91	
Total Lung Involvement Percent (n = 78)	n = 64	n = 14	n = 78	0.793
Mean ± SD	35.23 ± 23.08	44.58 ± 27.57	36.90 ± 24.03	
Median	40	49.50	40	
Comorbidities and Symptoms				
Sweating				0.025
No	487 (89.9%)	132 (97.1%)	619 (91.3%)	
Yes	55 (10.1%)	4 (2.9%)	59 (8.7%)	
Fever				0.183
No	264 (48.7%)	76 (55.9%)	340 (50.1%)	
Yes	278 (51.3%)	60 (44.1%)	338 (49.9%)	
Dyspnea				0.826
No	207 (38.2%)	50 (36.8%)	257 (37.9%)	
Yes	335 (61.8%)	86 (63.2%)	421 (62.1%)	
Chest Pain				0.073
No	454 (83.8%)	123 (90.4%)	577 (85.1%)	
Yes	88 (16.2%)	13 (9.6%)	101 (14.9%)	
Abnormal Lung Signs				< 0.0001
No	442 (81.5%)	91 (66.9%)	533 (78.6%)	
Yes	100 (18.5%)	45 (33.1%)	145 (21.4%)	
Diabetes				0.363
No	412 (76%)	102 (75%)	514 (75.8%)	
Yes	130 (24%)	34 (25%)	164 (24.2%)	
Hypertension				0.001
No	357 (65.9%)	71 (52.2%)	428 (63.1%)	
Yes	185 (34.1%)	61 (47.8%)	250 (36.9%)	
Cancers				< 0.0001
No	438 (80.8%)	83 (61%)	521 (76.8%)	
Yes	104 (19.2%)	53 (39%)	157 (23.2%)	
Cardiovascular Disease				0.006
No	376 (69.4%)	79 (58.1%)	455 (67.1%)	
Yes	166 (30.6%)	57 (41.9%)	223 (32.9%)	
CKD				< 0.0001
No	502 (92.6%)	94 (69.1%)	596 (87.9%)	
Yes	40 (7.4%)	42 (30.9%)	82 (12.1%)	
COPD				0.292
No	476 (87.8%)	115 (84.6%)	591 (87.2%)	
Yes	66 (12.2%)	21 (15.4%)	87 (12.8%)	
Treatment and Level of care				
Duration of Intubation (Day) (n = 37)	n = 7	n = 30	n = 37	< 0.0001
Mean ± SD	4.29 ± 4.07	5.87 ± 5.84	5.57 ± 5.53	
Median	3	4.50	4	
Duration of Non-invasive Ventilation (Day) (n = 30)	n = 17	n = 13	n = 30	0.054
Mean ± SD	5.82 ± 5.92	4.69 ± 3.66	5.33 ± 5.02	
Median	4	3	3.50	
Immunosuppressant Drugs				0.136
No	525 (96.9%)	135 (99.3%)	660 (97.3%)	
Yes	17 (3.1%)	1 (0.7%)	18 (2.7%)	
Anti-hypertensive Drugs				0.002
No	391 (72.1%)	82 (60.3%)	473 (69.8%)	
Yes	151 (27.9%)	54 (39.7%)	205 (30.2%)	
Pantoprazole				0.004
No	310 (57.2%)	60 (44.1%)	370 (54.6%)	
Yes	232 (42.8%)	76 (55.9%)	308 (45.4%)	
Hospitalization in a 14-day period prior to admission				0.001
No	456 (84.1%)	97 (71.3%)	553 (81.6%)	
Yes	86 (15.9%)	39 (28.7%)	125 (18.4%)	
ICU Admission				< 0.0001
No	428 (79%)	43 (31.6%)	471 (69.5%)	
Yes	112 (20.7%)	92 (67.6%)	204 (30.1%)	
Unknown	2 (0.4%)	1 (0.7%)	3 (0.4%)	

### Feature selection

Tables S4 and S5 (Additional file 1) show the details of the feature sets created for “at-admission” and “post-admission” death prediction based on the different feature selection methods. Features including cancers, CKD, oxygen saturation percent, BMI, age, hypertension, abnormal lung signs, and drug history were among the most prevalent features chosen by different feature selection methods. Furthermore, active smoking is considered important by many of our feature selection methods. According to our results, feature set 7 on “at admission” models and feature set 8 on “post admission models” had the best performance. The details of these feature sets are presented in Table 2.

**Table 2:** Best performing feature sets for “at admission” and “post admission” models

	Feature set	Method	Number of features	Features
**At admission**	7	Feature Importance using Gradient Boosting	20	Age, Oxygen Saturation Percent, Chronic Kidney Disease, Respiratory Rate, Diastolic Blood Pressure, Systolic Blood Pressure, BMI, Average Daily Used Cigarettes, Pantoprazole, Cancers, Hypertension, Abnormal Lung Signs, Drug History, Sex, Total Ling Involvement Percent, Hospitalization in a 14-day period prior to admission, Current Smoking, Cardiovascular Disease, Chronic Obstructive Pulmonary Disease, Diabetes
**Post admission**	8	Physician Opinion	24	Age, BMI, Systolic Blood Pressure, Diastolic Blood Pressure, Respiratory Rate, Oxygen Saturation Percent, Total Lung Involvement Percent, Sex, Current Smoking, History of Hookah consumption, Drug History, Fever, Dyspnea, Chest Pain, Diabetes, Hypertension, Cancers, Cardiovascular Disease, Chronic Kidney Disease, Chronic Obstructive Pulmonary Disease, Immunosuppressant Drugs, Duration of Intubation, Duration of Non-invasive ventilation, Admission in intensive care unit

### Model performance and evaluation

Details of our “at admission” models on different feature sets are reported in Table S6-S13 (Additional file 1). Comparing these models indicates that XGBoost outperformed the rest of the models in the majority of feature sets (except in feature set two which the random forest model outperformed the rest). Throughout feature sets, the weakest performance was for naive Bayes and logistic regression.

Tables S14-S21 (Additional file 1) present details of “post-admission models’ performance on different feature sets. The XGBoost outperformed the rest of the algorithms except for in feature set 6 which the ensemble model had better results. Furthermore, naive Bayes and logistic regression had the weakest performance throughout feature sets.

The probabilities of the top five models were calibrated. After calibration, accuracy, AUC, and F_1_ slightly decreased; however, logistic loss and brier score improved, showing improvement in the overall predictions of models.

**Fig. 2:**
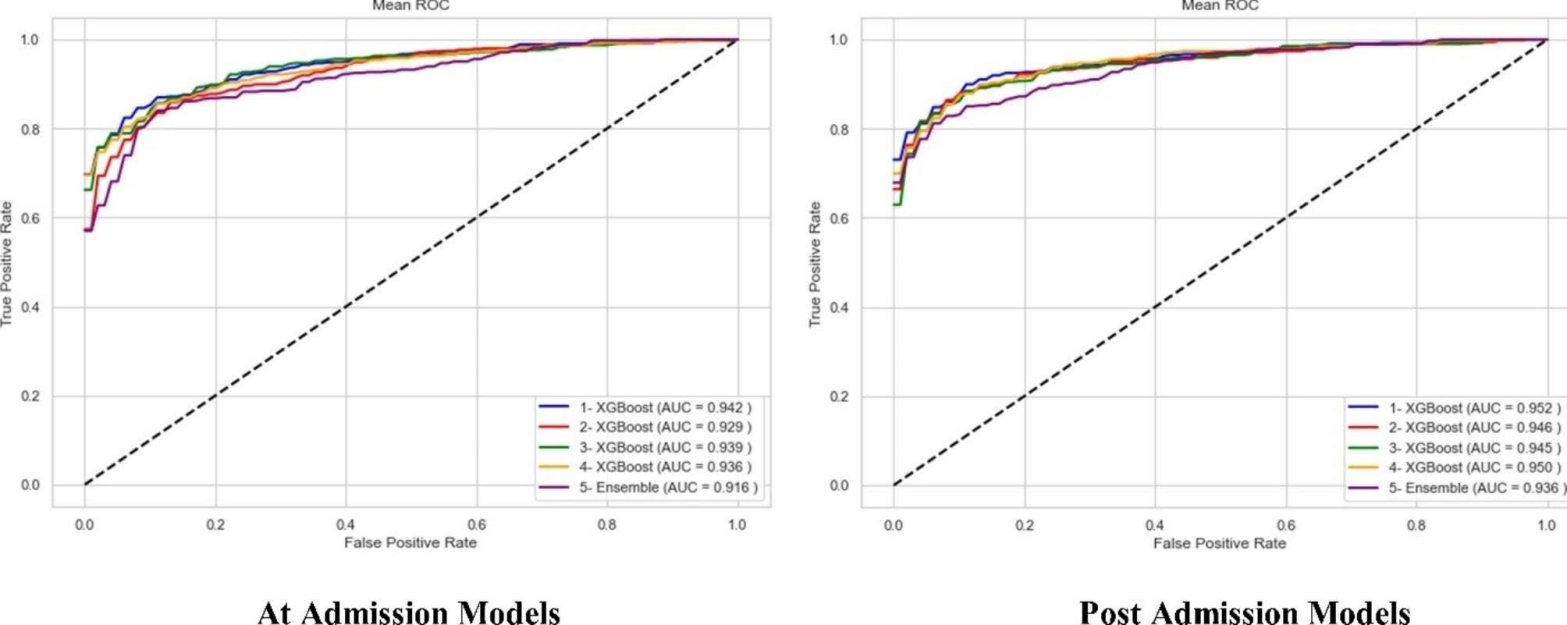
ROC AUC for the top “at admission” and “post admission” models

**Fig. 3:**
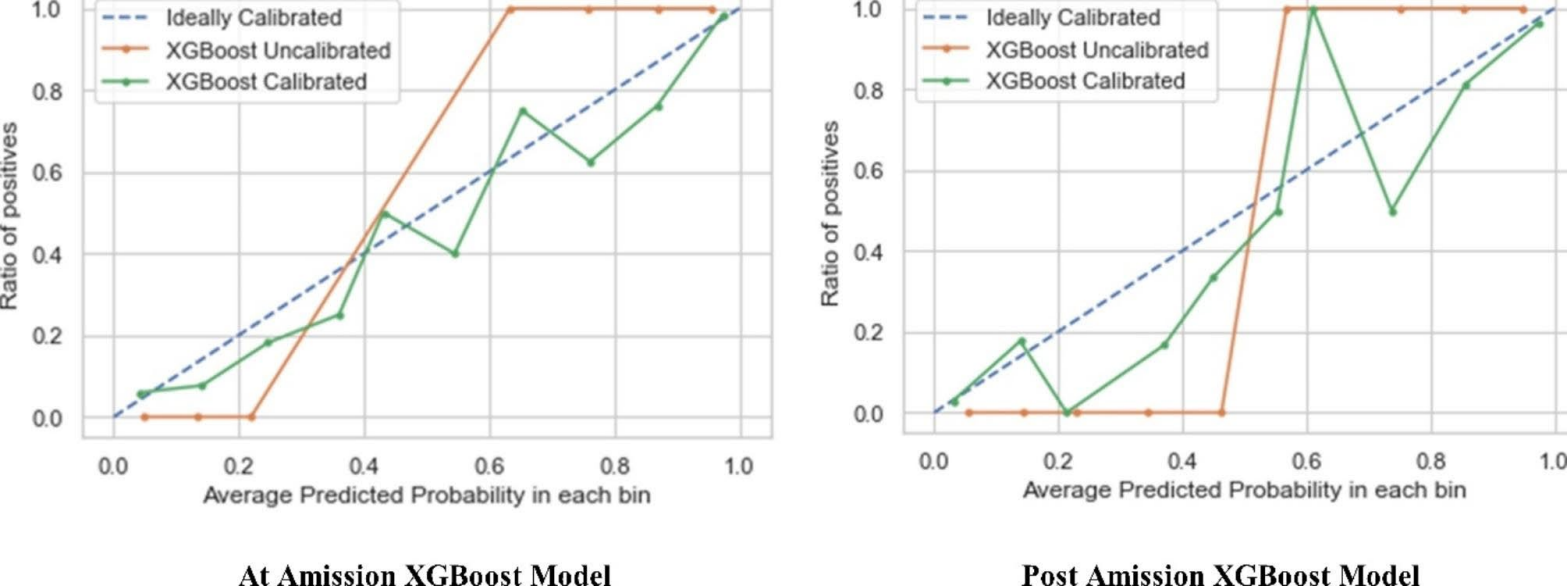
Calibration curve of the XGBoost model for “at admission” and “post admission” mortality prediction

The best “at admission” model was XGBoost which was trained using feature set seven (accuracy = 0.875, F_1_ score = 0.862). In addition, among “post admission” models, XGBoost trained on feature set eight (accuracy = 0.905, F_1_ score = 0.899) had the highest performance after calibration. Tables 3 and 4 report the performance of the top five calibrated and uncalibrated models. Figure 2 depicts the AUC of the top five “at admission” and “post admission” models. Figure 3 also shows the calibration curve for the best “at admission” and “post admission” XGBoost models.

**Table 3:** Performance results of top five “at admission” models

Rank	Algorithm	Feature set	Parameters	Calibration	Accuracy	AUC	Precision	Recall	F1 Score	Log Loss	Brier Score
1	XGBoost	7	Colsample_bytree = 0.3 Learning _rate = 0.01 n_estimators = 500 max_depth = 15	Uncalibrated	0.879	0.942	0.904	0.850	0.867	0.336	0.100
				Calibrated	0.875	0.940	0.904	0.839	0.862	0.310	0.094
2	XGBoost	8	Colsample_bytree = 0.5 Learning _rate = 0.01 n_estimators = 300 max_depth = 15	Uncalibrated	0.867	0.929	0.860	0.872	0.864	0.366	0.109
				Calibrated	0.859	0.927	0.870	0.837	0.849	0.329	0.100
3	XGBoost	5	Colsample_bytree = 0.3 Learning _rate = 0.01 n_estimators = 700 max_depth = 15	Uncalibrated	0.872	0.939	0.891	0.843	0.857	0.320	0.097
				Calibrated	0.864	0.938	0.895	0.819	0.843	0.314	0.096
4	XGBoost	3	Colsample_bytree = 0.3 Learning _rate = 0.01 n_estimators = 900 max_depth = 15	Uncalibrated	0.873	0.936	0.894	0.841	0.855	0.306	0.094
				Calibrated	0.872	0.934	0.906	0.824	0.849	0.316	0.095
5	Ensemble	5	XGBoost, MLP, Random Forest, Decision tree	Uncalibrated	0.860	0.916	0.870	0.842	0.850	0.361	0.113
				Calibrated	0.853	0.923	0.874	0.815	0.832	0.356	0.108

**Table 4:** Performance results of top five “post-admission” models

Rank	Algorithm	Feature set	Parameters	Calibration	Accuracy	AUC	Precision	Recall	F1 Score	Log Loss	Brier Score
1	XGBoost	8	Colsample_bytree = 0.3 Learning _rate = 0.01 n_estimators = 300 max_depth = 15	Uncalibrated	0.909	0.952	0.921	0.894	0.904	0.323	0.090
				Calibrated	0.905	0.951	0.921	0.885	0.899	0.246	0.072
2	XGBoost	5	Colsample_bytree = 0.3 Learning _rate = 0.1 n_estimators = 300 max_depth = 15	Uncalibrated	0.904	0.946	0.915	0.892	0.899	0.268	0.076
				Calibrated	0.902	0.943	0.917	0.885	0.896	0.282	0.079
3	XGBoost	3	Colsample_bytree = 0.5 Learning _rate = 0.1 n_estimators = 300 max_depth = 8	Uncalibrated	0.901	0.945	0.904	0.897	0.897	0.282	0.080
				Calibrated	0.897	0.944	0.904	0.886	0.892	0.288	0.081
4	XGBoost	7	Colsample_bytree = 0.5 Learning _rate = 0.01 n_estimators = 500 max_depth = 10	Uncalibrated	0.902	0.950	0.919	0.883	0.896	0.255	0.075
				Calibrated	0.897	0.948	0.916	0.872	0.889	0.266	0.076
5	Ensemble	8	XGBoost, SVM, Random Forest, Decision Tree, KNN	Uncalibrated	0.891	0.936	0.885	0.899	0.890	0.326	0.094
				Calibrated	0.885	0.935	0.894	0.870	0.879	0.292	0.086

### Feature importance

**Fig. 4:**
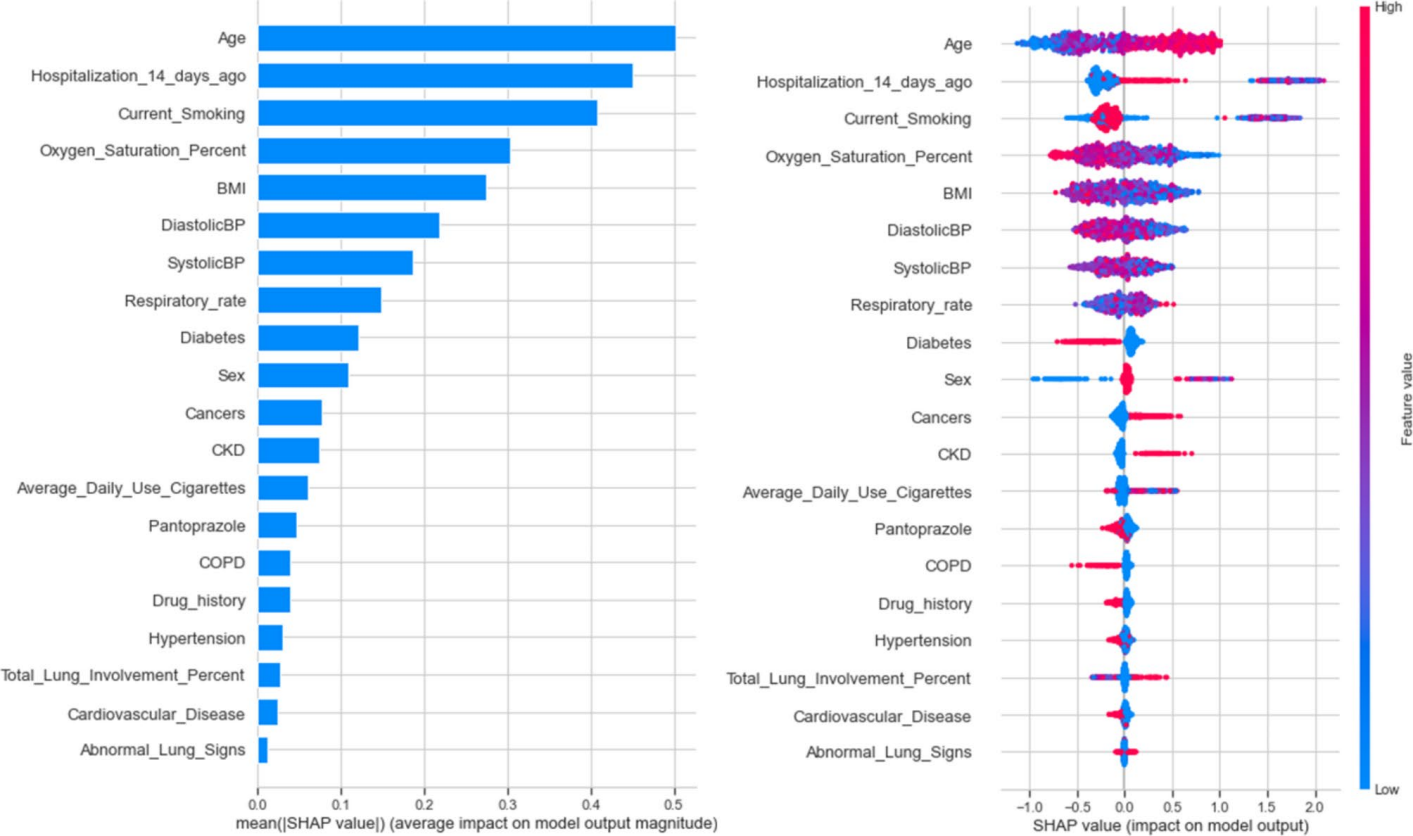
SHAP-based feature importance of “at admission” XGBoost model

Based on the SHAP method, in order, age, hospitalization in a 14-day period prior to admission, current smoking, SpO2%, BMI, diastolic and systolic blood pressure, respiratory rate, diabetes, and sex had the highest contribution in “at admission” mortality prediction. Figure 4 depicts the contribution of each feature to “at admission” XGBoost prediction model based on SHAP.

**Fig. 5:**
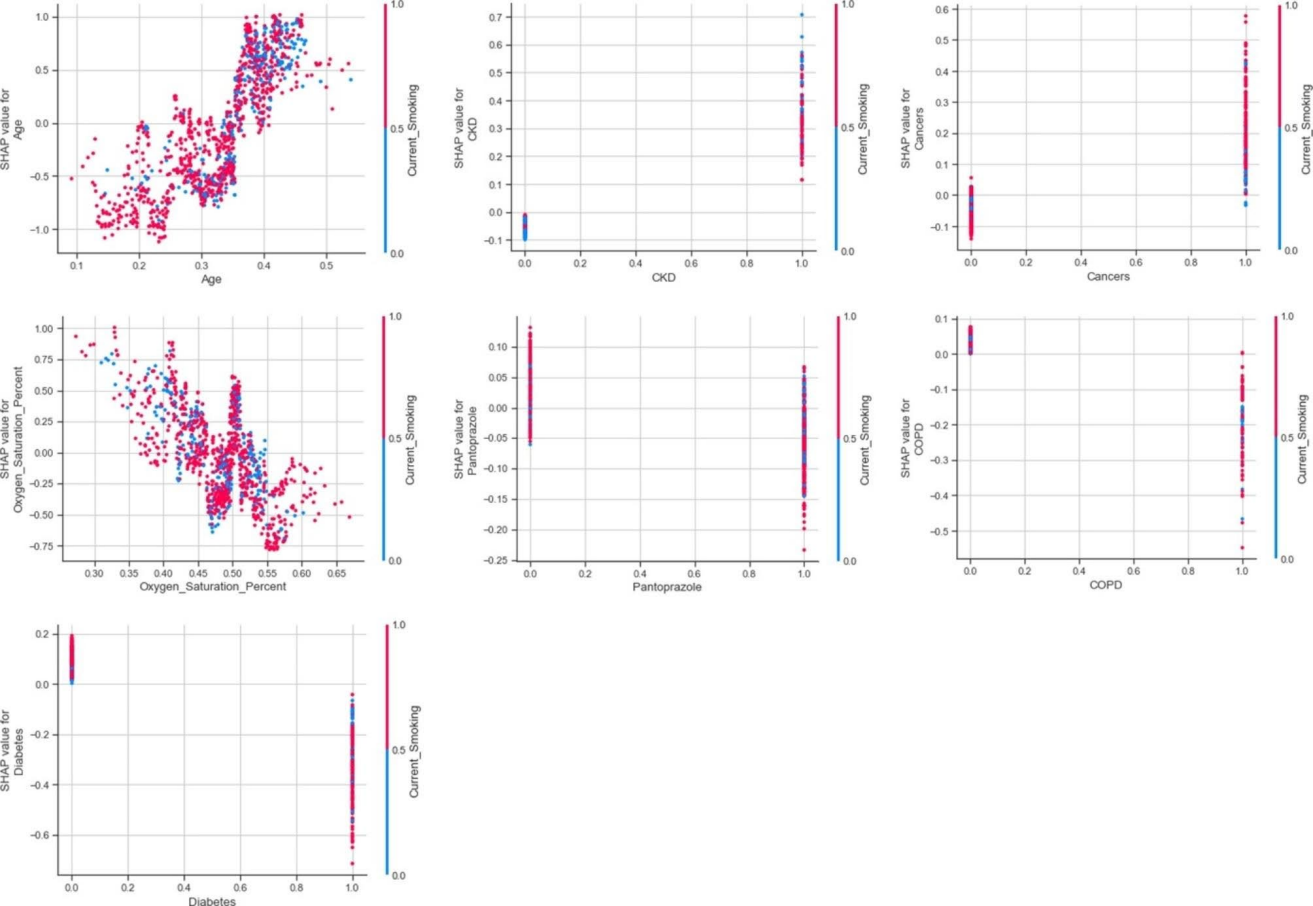
Current smoking SHAP dependence plots for at admission model

As presented in Fig. 5, older age, having cancer and CKD will lead to current smoking having higher SHAP value. While on the contrary, lower SpO2%, having diabetes, COPD and use of pantoprazole will result in lower SHAP value for current smokers. There are mixed effects for relationship between current smoking and other features (Figure S7, 2).

**Fig. 6:**
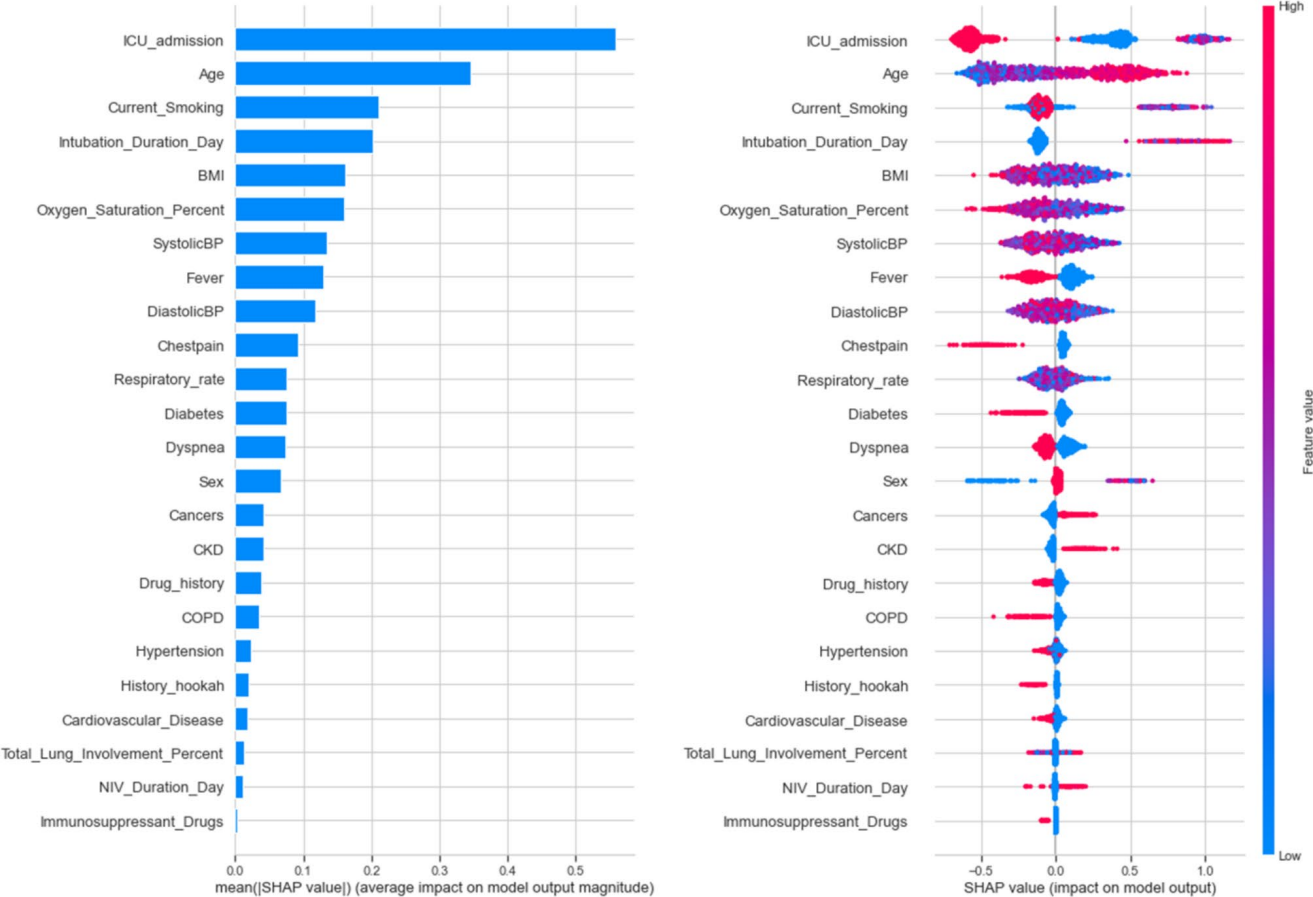
SHAP-based features importance of “post admission” XGBoost model

As presented in Fig. 6, admission in ICU, age, current smoking, duration of intubation, BMI, SpO2%, systolic blood pressure, fever, and diastolic blood pressure had the highest contribution to the “post admission” XGBoost model’s mortality prediction.

**Fig. 7:**
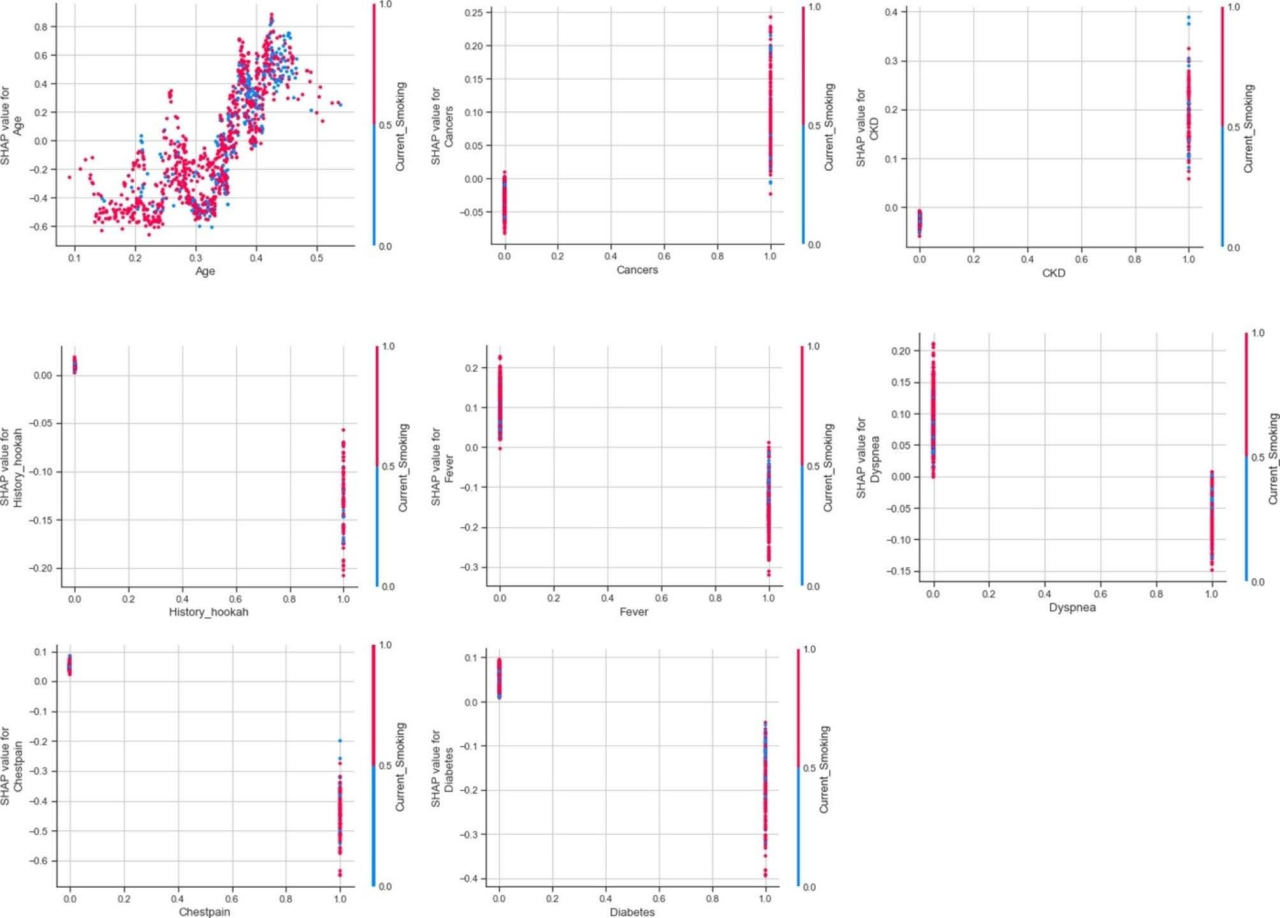
Current smoking SHAP dependence plots for post admission model

According to Fig. 7, older age, having cancer and CKD will lead to current smoking having higher SHAP value. While having fever, dyspnea, chest pain, diabetes and a history of hookah consumption will lead to current smoking having lower SHAP value. As presented in Figure S8 in 2, there are mixed effects for relationship between current smoking and other features.

### Error analysis

There were 140 errors in our “at admission” model, of which 52 cases were false positive, and 88 cases were false negative. Most of the errors were related to males (93.2%). Additionally, most of them had no COPD (76.7%), previous hospitalization (74.6%), diabetes (79.9%), drug history (72.4%), abnormal lung signs (75.4%), and CKD (76.1%).

There were also 103 errors in our “post admission” model of which 43 were false positive and 60 were false negative. Most of the cases were male (90.3%). The majority of these cases had no history of hookah consumption (92.2%), chest pain (93.2%), diabetes (77.7%), cancers (73.8%), CKD (75.7%), COPD (86.4%) and using immunosuppressant drugs (99%).

## Discussion

In the current study, multiple ML models were developed for the prediction of in-hospital mortality of COVID-19 patients with history of smoking. Furthermore, the models were evaluated and the highest-performing models for predicting patients’ chances of survival were identified.

Our results demonstrate that the best model for predicting mortality using patients’ information at admission is XGBoost (accuracy = 0.875, F_1_ score = 0.862) trained on the feature set seven (20 features). In addition, the best model for predicting mortality during hospitalization was also XGBoost (accuracy = 0.905, F_1_ score = 0.899) trained on the feature set eight (24 features). Naive Bayes and logistic regression performed substantially worse compared to XGBoost, random forest, and ensemble models.

These ML-based tools can assist clinicians and providers in patient triage [20, 61], resource allocation [26, 27], and providing the best possible care for patients [25, 62]. Input data of these models consist of patient demographics, comorbidities, medications, and levels of care that can be easily collected.

Results of the study suggest that active smoking, age, sex, ICU admission, hospitalization in a 14-day period prior to admission, SpO2%, duration of intubation, BMI, diastolic and systolic blood pressure, fever, respiratory rate, diabetes, CKD, COPD, cancers and drug history were among most important predictors of COVID-19 mortality. This is in line with previous studies which showed that age, sex, oxygen saturation, diabetes, use of opioids, respiratory diseases, CKD, and cancers could increase mortality [63–67]. Another study similarly indicated that age and SpO2% are independent markers of survival in COVID-19 patients [68]. Moreover, SpO2% was identified as an important feature in predicting in-hospital mortality in another study [69]. Yanyan et al. [70] indicated that age, sex, and diabetes are important mortality risk factors in COVID-19 patients, which is in accordance with our results. These studies are not specifically on smokers; therefore, it can be concluded that these are important risk factors among both smokers and non-smokers.

In contrast to previous studies suggesting a lack of association between prior smoking history and mortality in COVID-19 patients [71–73] or potential protective effects [74, 75], our results indicate that smoking is an important risk factor in COVID-19 mortality. This was according to previous studies which believed smoking is an important risk factor of mortality due to impairment of lung and respiratory diseases [9–13].

Based on our results, active smoking was among the most important features in predicting mortality (the third most important feature in both models). Salah et al. [76] suggest that patients which were either active smokers or former smokers have a higher mortality risk and patients that are active smokers have twice the mortality risk compared to those who were former smokers. Bellan et al. [11], using cohort data from Italian patients, identified smoking as an independent mortality predictor in COVID-19 patients. A meta-analysis [77], which included 60 studies and 51,225 patients from 13 countries, found smoking was one of the major predictors of mortality in COVID-19 patients. Parra-Bracamonte et al. [78], after analyzing a huge dataset from Mexico, found that smoking was not a risk factor for mortality. Our results indicate that active smoking may have a mixed effect on mortality. According to Figs. 4 and 6, in some cases, active smoking contributes to the mortality of patients and in some cases, it does not have such a contribution. Thus, further research is needed to prove the role of smoking in patient mortality.

Kar et al. [79] developed a COVID-19 prediction model for patients at admission using retrospective cohort data. However, their model had a higher accuracy (97%) than our model which could be due to their greater sample size (2370 patients). In addition, they did not consider smokers. Fink et al. [80] developed a prediction model using data from 24 h after admission. Our best models outperformed their model (AUC = 0.85). In a previous study [68], the mortality prediction model reached an accuracy of 89% and an AUC of 86%, which is lower than our best models. Our models also outperform another in-hospital mortality prediction model which was developed by Shiri et al. [69]. Using the XGBoost algorithm and demographic, clinical, imaging, and laboratory results, they were able to achieve 88% accuracy, which was lower than our post-admission model. However, they did not use features relating to smoking and opioid use in their models.

### Limitations

Due to the small number of patients that have a history of smoking registered in our database, we were not able to perform external validation. Furthermore, due to our small sample size, we could not train separate models for different subpopulations such as age groups. Future studies are necessary for developing models to predict mortality in smoking COVID-19 patients for different age groups and levels of care. Some of the features that were identified as important predictors of COVID-19 mortality had high missing rates (including BMI, hospitalization in a 14-day period prior to admission, respiratory rate, and systolic and diastolic blood pressure), thus further studies are needed to investigate the role of these features on patient mortality.

## Conclusion

In the present study, multiple mortality predictive models were developed and evaluated for use at admission and after admission during patients’ stay in hospitals. The best-calibrated models for admission and post-admission are XGBoost (accuracy = 0.875, F_1_ score = 0.862) and XGBoost (accuracy = 0.905, F_1_ score = 0.899), respectively. Additionally, the current study reported the explainability of models in terms of SHAP-based feature importance that identified variables strongly associated with mortality. Previous studies indicate that mortality prediction models have some biases for subpopulations that have different risks of mortality, such as smokers [20]. The current study demonstrates the potential of ML-based predictive models for quantification pre and post-admission COVID-19 mortality rates, facilitating effective decision making in management of patients with history of smoking.

## Electronic supplementary material

Below is the link to the electronic supplementary material.

Supplementary Material 1

Supplementary Material 2

## Data Availability

Data sharing is not applicable to this study, due to the restrictions by the ethics research committee of Tehran University of Medical Sciences (TUMS) which have been regulated in order to protect patient privacy. Corresponding author should be contacted if there is a reasonable request for data. The code used for this study is available from the GitHub repository (https://github.com/ali-sharifikia/covid19-mortality-prediction).

## List of Abbreviations

ML: Machine Learning
SHAP: Shapely Additive Explanation
COVID-19: Corona Virus 2019
AUC: Area Under Curve
CKD: Chronic Kidney Disease
COPD: Chronic Obstructive Pulmonary Disease
BMI: Body Mass Index
SpO2%: Oxygen Saturation Percent
ICU: Intensive Care Unit
SMOTE: Synthetic minority oversampling technique
