# Supplementary material for: Machine learning-based mortality prediction models for smoker COVID-19 patients
Source: BMC Med Inform Decis Mak. 2023 Jul 21;23:129. doi: 10.1186/s12911-023-02237-w (PMC10360290; doi:10.1186/s12911-023-02237-w)
Supplement: Supplementary file 1 — Supplementary Material 1 [file 12911_2023_2237_MOESM1_ESM.docx]

Supplementary data (Tables)

Content

[Table S1 List of variables in the dataset collected from the registry 2](#_Toc127641327)

[Table S2. Missing rate of features 4](#_Toc127641328)

[Table S3. Performance of models before oversampling 5](#_Toc127641329)

[Table S4. Feature sets created for models developed for death prediction at admission 6](#_Toc127641330)

[Table S5*.* Feature sets created for models developed for death prediction after admission 7](#_Toc127641331)

[Table S6. Performance of “at admission” models on feature set 1 8](#_Toc127641332)

[Table S7. Performance of “at admission” models on feature set 2 9](#_Toc127641333)

[Table S8. Performance of “at admission” models on feature set 3 10](#_Toc127641334)

[Table S9. Performance of “at admission” models on feature set 4 11](#_Toc127641335)

[Table S10. Performance of “at admission” models on feature set 5 12](#_Toc127641336)

[Table S11. Performance of “at admission” models on feature set 6 13](#_Toc127641337)

[Table S12. Performance of “at admission” models on feature set 7 14](#_Toc127641338)

[Table S13. Performance of “at admission” models on feature set 8 15](#_Toc127641339)

[Table S14. Performance of “post admission” models on feature set 1 16](#_Toc127641340)

[Table S15. Performance of “post admission” models on feature set 2 17](#_Toc127641341)

[Table S16. Performance of “post admission” models on feature set 3 18](#_Toc127641342)

[Table S17. Performance of “post admission” models on feature set 4 19](#_Toc127641343)

[Table S18. Performance of “post admission” models on feature set 5 20](#_Toc127641344)

[Table S19. Performance of “post admission” models on feature set 6 21](#_Toc127641345)

[Table S20. Performance of “post admission” models on feature set 7 22](#_Toc127641346)

[Table S21. Performance of “post admission” models on feature set 8 23](#_Toc127641347)

Table S1 List of variables in the dataset collected from the registry

| **#** | **Feature** | **#** | **Feature** |
| --- | --- | --- | --- |
| 1 | Age | 93 | Chronic Liver Disease severity |
| 2 | Weight | 94 | Rheumatological diseases |
| 3 | Height | 95 | Hypertension |
| 4 | Body Mass Index (BMI) | 96 | Hyperlipidemia |
| 5 | Length of Stay (LOS) | 97 | Hypothyroidism |
| 6 | Onset to symptom time | 98 | Immune deficiency disease |
| 7 | Average Daily Use Cigarettes | 99 | Neurological diseases |
| 8 | Hookah Consumption | 100 | Non-Invasive Ventilation |
| 9 | Temp | 101 | Heparin |
| 10 | Systolic Blood Pressure | 102 | Diabetic Drugs |
| 11 | Diastolic Blood Pressure | 103 | Rheumatological diseases type |
| 12 | Respiratory rate | 104 | Neurological diseases type |
| 13 | Oxygen Saturation Percent | 105 | Metastasis status |
| 14 | Alkaline Phosphatase (ALP) | 106 | Metoral |
| 15 | Alanine Transaminase (ALT) | 107 | Losartan |
| 16 | Aspartate Transaminase (AST) | 108 | History smoking |
| 17 | Albumin | 109 | History hookah |
| 18 | Blood Sugar | 110 | Fat lowering drugs |
| 19 | Creatine Phosphokinase (CPK) | 111 | Fatty Liver |
| 20 | C-reactive Protein (CRP) | 112 | Human Immunodeficiency Virus (HIV) |
| 21 | Calcium | 113 | Enalapril |
| 22 | Creatinine | 114 | Total Lung Involvement Rank |
| 23 | D dimer | 115 | Pericardial Effusion |
| 24 | Direct bilirubin | 116 | Diuretic |
| 25 | Erythrocyte Sedimentation Rate (ESR) | 117 | Hemodialysis |
| 26 | Fasting Blood Sugar (FBS) | 118 | Corticosteroid pulse therapy |
| 27 | Ferritin | 119 | Hemoperfusion |
| 28 | Hemoglobin (HB) | 120 | High Respiratory Rate |
| 29 | Lactate Dehydrogenase (LDH) | 121 | Hypoxemia |
| 30 | Lymphocyte | 122 | Liver Transplantation |
| 31 | Weakness and lethargy duration | 123 | Kidney Transplantation |
| 32 | Magnesium (Mg) | 124 | Chronic Obstructive Pulmonary Disease |
| 33 | Neutrophil | 125 | Asthma |
| 34 | Platelet Count (PLT) | 126 | Asprin Consumption |
| 35 | PROBNP | 127 | Wet Cough |
| 36 | Phosphor | 128 | Weight Loss |
| 37 | Potassium | 129 | Weakness and Lethargy |
| 38 | Procalcitonin | 130 | Vomit |
| 39 | Sodium | 131 | Trembling |
| 40 | Total bilirubin | 132 | Sweating |
| 41 | Troponin | 133 | Sputum |
| 42 | Urea | 134 | Sore throat |
| 43 | Venous blood gas (VBG) | 135 | Rush |
| 44 | HCO3 | 136 | Rhinorrhea |
| 45 | PCO2 | 137 | Nausea |
| 46 | PH | 138 | Muscle Pain |
| 47 | PO2 | 139 | Lymphadenopathy |
| 48 | SO2 | 140 | Loss of taste |
| 49 | White Blood Cells (WBC) | 141 | Loss of smell |
| 50 | PSO2_on_ Admission | 142 | Loss of Consciousness |
| 51 | Intubation duration (Day) | 143 | Limb Edema |
| 52 | Intensive Care Unit Length of Stay (ICU_LOS) | 144 | Joint pain (Arthralgia) |
| 53 | Total Lung Involvement Percent | 145 | Hemoptysis |
| 54 | Ejection fraction rate | 146 | Headache |
| 55 | Vomit duration | 147 | Fever |
| 56 | Body Pain duration | 148 | Fatigue |
| 57 | Fever duration | 149 | Epigastric |
| 58 | Heavy Breathing duration | 150 | Dyspnea |
| 59 | Nausea duration | 151 | Dry Cough |
| 60 | Headache duration | 152 | Dizziness |
| 61 | Dry cough duration | 153 | Diarrhea |
| 62 | Non-Invasive Ventilation Duration (Day) | 154 | Chest pain |
| 63 | Sex | 155 | Cardiac Arrhythmia |
| 64 | Prednisolone | 156 | Body Pain |
| 65 | Hydroxychloroquine | 157 | Bleeding |
| 66 | Naproxen | 158 | Ataxia |
| 67 | Chronic Kidney Disease Type | 159 | Anorexia |
| 68 | Cardiac disease type | 160 | Abnormal Lung Signs |
| 69 | Cerebrovascular Accident type | 161 | O2_insection |
| 70 | Stage of care | 162 | Non-Invasive Ventilation Simple Mask in section |
| 71 | Covid drug history | 163 | Intubation at section |
| 72 | Gastric Ulcer | 164 | Interferon a1b1 |
| 73 | Dexamethasone history | 165 | Intravenous immune globulin (IVIG) |
| 74 | Severe obesity | 166 | Corticosteroids treatment |
| 75 | Peripheral Arterial Disease (PAD) | 167 | Corticosteroid history |
| 76 | Diabetes other organ involvement | 168 | Cancers |
| 77 | Azithromycin | 169 | Captopril |
| 78 | Drug history | 170 | Cardiovascular Disease |
| 79 | Alcohol history | 171 | Chronic Kidney Disease |
| 80 | Chronic heart disease type | 172 | Chronic Liver Disease |
| 81 | Diabetes | 173 | Blood Pressure Drugs |
| 82 | Dementia | 174 | Other Anti-hypertensive Drugs |
| 83 | Current Hookah | 175 | Cerebrovascular Accident (CVA) |
| 84 | Current Smoking | 176 | Plasmapheresis |
| 85 | Intensive Care Unit admission | 177 | Pantoprazole |
| 86 | Intubation in section | 178 | Oseltamivir |
| 87 | Intubation in Intensive Care Unit | 179 | Hospitalization 14 days ago |
| 88 | Metastatic status | 180 | Tumor Grade |
| 89 | Transplantation | 181 | Cancer Stage |
| 90 | Sugar lowering drugs | 182 | Aspirin Consumption at Hospital |
| 91 | Kidney disease severity | 183 | Death |
| 92 | Schizophrenia |  |  |

Table S2. Missing rate of features

| # | Feature | Missing percentage |
| --- | --- | --- |
| 1 | Age | 1.18 |
| 2 | BMI | 20.71 |
| 3 | Systolic Blood Pressure | 10.32 |
| 4 | Diastolic Blood Pressure | 12.54 |
| 5 | Respiratory Rate | 17.70 |
| 6 | Oxygen Saturation Percent | 6.49 |
| 7 | Total Lung Involvement Percent | 1.18 |
| 8 | Sex | 0.15 |
| 9 | Current Smoking | 7.64 |
| 10 | Hospitalization 14 Days Ago | 21.42 |
| 11 | Admission in Intensive Care Unit | 1.47 |

Table S3. Performance of models before oversampling

| **Feature set** | **Accuracy** | **AUC** | **Precision** | **Recall** | **F1 Score** | **Log-Loss** |
| --- | --- | --- | --- | --- | --- | --- |
| 1 | 0.751 | 0.688 | 0.355 | 0.287 | 0.311 | 0.981 |
| 2 | 0.714 | 0.572 | 0.296 | 0.263 | 0.263 | 1.273 |
| 3 | 0.761 | 0.691 | 0.346 | 0.263 | 0.294 | 0.890 |
| 4 | 0.767 | 0.684 | 0.412 | 0.302 | 0.340 | 0.943 |
| 5 | 0.782 | 0.701 | 0.436 | 0.294 | 0.344 | 0.895 |
| 6 | 0.783 | 0.705 | 0.446 | 0.287 | 0.341 | 0.886 |
| 7 | 0.788 | 0.743 | 0.426 | 0.293 | 0.342 | 0.776 |
| 8 | 0.791 | 0.727 | 0.453 | 0.315 | 0.360 | 0.807 |

Table S4. Feature sets created for models developed for death prediction at admission

| Feature set | Method | Number of features | Features |
| --- | --- | --- | --- |
| 1 | Statistical analysis | 17 | Age, Oxygen Saturation Percent, Sweating, Abnormal Lung Signs, Chronic Kidney Disease, Hospitalized 14 days ago, BMI, Sex, Current Smoking, Fever, Chest Pain, Hypertension, Cancers, Cardiovascular Disease, Immunosuppressant Drugs, Antihypertensive Drugs, Pantoprazole |
| 2 | RFECV using Logistic Regression | 20 | Abnormal Lung Signs, Antihypertensive Drugs, Chronic Kidney Disease, Chronic Obstructive Pulmonary Disease, Cancers, Cardiovascular Disease, Chest Pain, Current Smoking, Diabetes, Drug History, Dyspnea, Fever, History Hookah, Hospitalization 14 days ago, Hypertension, Immunosuppressant Drugs, Oxygen Saturation Percent, Pantoprazole, Sex, Sweating |
| 3 | RFECV using Random Forest | 27 | Abnormal Lung Signs, Age, Antihypertensive Drugs, Average Daily Used Cigarettes, BMI, Chronic Kidney Disease, Chronic Obstructive Pulmonary Disease, Cancers, Cardiovascular Disease, Chest Pain, Current Smoking, Diabetes, Diastolic Blood Pressure, Drug History, Dyspnea, Fever, History Hookah, Hospitalization 14 days ago, Hypertension, Immunosuppressant Drugs, Oxygen Saturation Percent, Pantoprazole, Respiratory Rate, Sex, Sweating, Systolic Blood Pressure, Total Lung Involvement Percent |
| 4 | RFECV using Gradient Boosting | 5 | Age, Chronic Kidney Disease, Cancers, Diastolic Blood Pressure, Oxygen Saturation Percent |
| 5 | Feature Importance using Extra trees classifier | 20 | Age, Oxygen Saturation Percent, Chronic Kidney Disease, Respiratory Rate, Diastolic Blood Pressure, Systolic Blood Pressure, BMI, Cancers, Average Daily Used Cigarettes, Fever, Pantoprazole, Abnormal Lung Signs, Drug History, Current Smoking, Diabetes, Cardiovascular Disease, Dyspnea, Hospitalization 14 days ago, Hypertension, Antihypertensive drug |
| 6 | Feature importance using Random Forest | 12 | Age, Chronic Kidney Disease, Oxygen Saturation Percent, Diastolic Blood Pressure, BMI, Hypertension, Cancers, Cardiovascular Disease, Hospitalization 14 days ago, Abnormal Lung Signs, Average Daily Used Cigarettes, Pantoprazole |
| 7 | Feature Importance using Gradient Boosting | 20 | Age, Oxygen Saturation Percent, Chronic Kidney Disease, Respiratory Rate, Diastolic Blood Pressure, Systolic Blood Pressure, BMI, Average Daily Used Cigarettes, Pantoprazole, Cancers, Hypertension, Abnormal Lung Signs, Drug History, Sex, Total Ling Involvement Percent, Hospitalization 14 days ago, Current Smoking, Cardiovascular Disease, Chronic Obstructive Pulmonary Disease, Diabetes |
| 8 | Physician Opinion | 21 | Age, BMI, Systolic Blood Pressure, Diastolic Blood Pressure, Respiratory Rate, Oxygen Saturation Percent, Total Lung Involvement Percent, Sex, Current Smoking, History of Hookah consumption, Drug History, Fever, Dyspnea, Chest Pain, Diabetes, Hypertension, Cancers, Cardiovascular Disease, Chronic Kidney Disease, Chronic Obstructive Pulmonary Disease, Immunosuppressant Drugs |

Table S5*.* Feature sets created for models developed for death prediction after admission

| **Feature set** | **Method** | **Number of features** | **Features** |
| --- | --- | --- | --- |
| 1 | Statistical analysis | 20 | Age, Oxygen Saturation Percent, Sweating, Abnormal Lung Signs, Chronic Kidney Disease, Hospitalized 14 days ago, BMI, Sex, Current Smoking, Fever, Chest Pain, Hypertension, Cancers, Cardiovascular Disease, Immunosuppressant Drugs, Antihypertensive Drugs, Pantoprazole, Duration of Intubation, Duration of Non-invasive ventilation, Admission in intensive care unit |
| 2 | RFECV using Logistic Regression | 25 | Age, Abnormal Lung Signs, Antihypertensive Drugs, BMI, Chronic Kidney Disease, Chronic Obstructive Pulmonary Disease, Cancers, Cardiovascular Disease, Chest Pain, Current Smoking, Diabetes, Drug History, Dyspnea, Fever, History of Hookah consumption, Hospitalized 14 days ago, Hypertension, Admission in intensive care unit, Immunosuppressant Drugs, Duration of Intubation, Duration of Non-invasive ventilation, Oxygen Saturation Percent, Pantoprazole, Sex, Sweating |
| 3 | RFECV using Random Forest | 18 | Age, Abnormal Lung Signs, Average Daily Used Cigarettes, BMI, Chronic Kidney Disease, Cancers, Diastolic Blood Pressure, Drug history, Dyspnea, Fever, Hospitalized 14 days ago, Hypertension, Admission in intensive care unit, Duration of Intubation, Oxygen Saturation Percent, Pantoprazole, Respiratory Rate, Systolic Blood Pressure |
| 4 | RFECV using Gradient Boosting | 4 | Age, Chronic Kidney Disease, Admission in intensive care unit, Duration of Intubation |
| 5 | Feature Importance using Extra trees classifier | 20 | Admission in intensive care unit, Age, Oxygen Saturation Percent, Chronic Kidney Disease, Diastolic Blood Pressure, Respiratory Rate, Systolic Blood Pressure, Duration of Intubation, BMI, Cancers, Dyspnea, Drug History, Pantoprazole, Abnormal Lung Signs, Fever, Cardiovascular Disease, Antihypertensive Drugs, Hypertension, Hospitalized 14 days ago, Average Daily Used Cigarettes |
| 6 | Feature importance using Random Forest | 16 | Duration of Intubation, Cancers, Chronic Kidney Disease, Admission in intensive care unit, Diastolic Blood Pressure, Systolic Blood Pressure, Oxygen Saturation Percent, Hospitalized 14 days ago, Chest Pain, Antihypertensive Drugs, Sweating, BMI, Cardiovascular Disease, Pantoprazole, Current Smoking, Average Daily Used Cigarettes |
| 7 | Feature Importance using Gradient Boosting | 20 | Admission in intensive care unit, Age, Duration of Intubation, Oxygen Saturation Percent, Chronic Kidney Disease, Cancers, Respiratory Rate, Systolic Blood Pressure, Diastolic Blood Pressure, BMI, Average Daily Used Cigarettes, Drug History, Abnormal Lung Signs, COPD, Sweating, Total Lung Involvement Percent, Chest Pain, Cardiovascular Disease, Sex, Hospitalized 14 days ago |
| 8 | Physician Opinion | 24 | Age, BMI, Systolic Blood Pressure, Diastolic Blood Pressure, Respiratory Rate, Oxygen Saturation Percent, Total Lung Involvement Percent, Sex, Current Smoking, History of Hookah consumption, Drug History, Fever, Dyspnea, Chest Pain, Diabetes, Hypertension, Cancers, Cardiovascular Disease, Chronic Kidney Disease, Chronic Obstructive Pulmonary Disease, Immunosuppressant Drugs, Duration of Intubation, Duration of Non-invasive ventilation, Admission in intensive care unit |

Table S6. Performance of “at admission” models on feature set 1

| **Algorithm** | **Parameters** | **Accuracy** | **AUC** | **Precision** | **Recall** | **F1 Score** | **Log Loss** | **Brier Score** |
| --- | --- | --- | --- | --- | --- | --- | --- | --- |
| XGBoost | Colsample_bytree = 0.8  Learning _rate = 0.1  n_estimators = 100  max_depth = 15 | 0.841 | 0.914 | 0.849 | 0.815 | 0.824 | 0.367 | 0.117 |
| SVM | C = 100  Gamma = 1  Kernel = rbf | 0.777 | 0.848 | 0.782 | 0.749 | 0.759 | 0.475 | 0.155 |
| MLP | Activation = relu  Alpha = 0.05  Hidden_layer_sizes = 15  Learning_rate = constant  Solver = lbfgs | 0.784 | 0.823 | 0.769 | 0.793 | 0.777 | 1.281 | 0.185 |
| KNN | Metric = manhattan  Weights = distance  N_neighbours = 8 | 0.750 | 0.809 | 0.732 | 0.778 | 0.752 | 0.765 | 0.178 |
| Decision Tree | Max_depth = 8  Criterion = entropy  Min_samples_leaf = 3  Min_samples_split = 12 | 0.784 | 0.851 | 0.789 | 0.767 | 0.771 | 1.805 | 0.158 |
| Random Forest | Max_depth = 16  N_estimators = 700  Min_samples_leaf = 3  Min_samples_split = 8 | 0.829 | 0.902 | 0.826 | 0.820 | 0.819 | 0.402 | 0.126 |
| Logistic regression | C = 100  Penalty = l1  Solver = liblinear | 0.709 | 0.779 | 0.690 | 0.759 | 0.772 | 0.577 | 0.191 |
| Naive bayes | Var_smoothing = 0.0001 | 0.651 | 0.752 | 0.597 | 0.949 | 0.732 | 1.769 | 0.318 |
| Ensemble | XGBoost, Random Forest, Decision Tree, KNN, Logistic Regression | 0.833 | 0.903 | 0.826 | 0.830 | 0.821 | 0.402 | 0.125 |

Results of our models on feature set 1 demonstrate that best performance was for XGBoost model (accuracy = 0.841, F1 = 0.821) and naive Bayes was the poorest (accuracy = 0.651, F1 = 0.732).

Table S7. Performance of “at admission” models on feature set 2

| **Algorithm** | **Parameters** | **Accuracy** | **AUC** | **Precision** | **Recall** | **F1 Score** | **Log Loss** | **Brier Score** |
| --- | --- | --- | --- | --- | --- | --- | --- | --- |
| XGBoost | Colsample_bytree = 0.5  Learning _rate = 0.001  n_estimators = 900  max_depth = 10 | 0.821 | 0.877 | 0.845 | 0.753 | 0.779 | 0.529 | 0.172 |
| SVM | C = 100  Gamma = 0.1  Kernel = rbf | 0.732 | 0.801 | 0.708 | 0.771 | 0.736 | 0.548 | 0.180 |
| MLP | Activation = tanh  Alpha = 0.0001  Hidden_layer_sizes = (15,10)  Learning_rate = constant  Solver = adam | 0.754 | 0.818 | 0.734 | 0.778 | 0.752 | 0.916 | 0.193 |
| KNN | Metric = manhattan  Weights = distance  N_neighbours = 6 | 0.728 | 0.793 | 0.689 | 0.822 | 0.748 | 1.517 | 0.196 |
| Decision Tree | Max_depth = 8  Criterion = gini  Min_samples_leaf = 4  Min_samples_split = 8 | 0.790 | 0.830 | 0.798 | 0.740 | 0.758 | 2.303 | 0.169 |
| Random Forest | Max_depth = 32  N_estimators = 700  Min_samples_leaf = 3  Min_samples_split = 10 | 0.816 | 0.875 | 0.815 | 0.788 | 0.791 | 0.438 | 0.140 |
| Logistic regression | C = 10  Penalty = l2  Solver = newton-cg | 0.722 | 0.770 | 0.694 | 0.774 | 0.730 | 0.586 | 0.194 |
| Naive bayes | Var_smoothing = 0.08111308307896872 | 0.675 | 0.756 | 0.619 | 0.905 | 0.735 | 1.889 | 0.303 |
| Ensemble | XGBoost, SVM, MLP,Random Forest, Decision Tree, KNN, Logistic Regression, Naïve Bayes | 0.812 | 0.878 | 0.817 | 0.766 | 0.776 | 0.477 | 0.151 |

For feature set 2, highest and lowest performance were for random forest (accuracy = 0.816, F1 = 0.791) and logistic regression (accuracy = 0.722, F1 = 0.730), respectively.

Table S8. Performance of “at admission” models on feature set 3

| Algorithm | Parameters | Accuracy | AUC | Precision | Recall | F1 Score | Log Loss | Brier Score |
| --- | --- | --- | --- | --- | --- | --- | --- | --- |
| XGBoost | Colsample_bytree = 0.3  Learning _rate = 0.01  n_estimators = 900  max_depth = 15 | 0.873 | 0.936 | 0.894 | 0.841 | 0.855 | 0.306 | 0.094 |
| SVM | C = 100  Gamma = 1  Kernel = rbf | 0.781 | 0.847 | 0.778 | 0.763 | 0.761 | 0.484 | 0.158 |
| MLP | Activation = relu  Alpha = 0.05  Hidden_layer_sizes = 10  Learning_rate = constant  Solver = lbfgs | 0.771 | 0.821 | 0.749 | 0.798 | 0.767 | 1.404 | 0.200 |
| KNN | Metric = manhattan  Weights = distance  N_neighbours = 6 | 0.735 | 0.825 | 0.688 | 0.857 | 0.762 | 1.312 | 0.181 |
| Decision Tree | Max_depth = 8  Criterion = gini  Min_samples_leaf = 3  Min_samples_split = 10 | 0.800 | 0.850 | 0.796 | 0.796 | 0.789 | 2.235 | 0.161 |
| Random Forest | Max_depth = 16  N_estimators = 700  Min_samples_leaf = 3  Min_samples_split = 10 | 0.849 | 0.919 | 0.855 | 0.832 | 0.837 | 0.398 | 0.121 |
| Logistic regression | C = 10  Penalty = l2  Solver = newton-cg | 0.734 | 0.798 | 0.714 | 0.770 | 0.739 | 0.557 | 0.182 |
| Naive bayes | Var_smoothing = 0.012328467394420659 | 0.678 | 0.769 | 0.622 | 0.907 | 0.737 | 1.711 | 0.294 |
| Ensemble | XGBoost, MLP, Random Forest, Decision Tree | 0.852 | 0.927 | 0.862 | 0.833 | 0.837 | 0.347 | 0.108 |

As for feature set 3, best model was XGBoost (accuracy = 0.873, F1 = 0.855) and the poorest one was naive Bayes (accuracy = 0.678, F1 = 0.737).

Table S9. Performance of “at admission” models on feature set 4

| Algorithm | Parameters | Accuracy | AUC | Precision | Recall | F1 Score | Log Loss | Brier Score |
| --- | --- | --- | --- | --- | --- | --- | --- | --- |
| XGBoost | Colsample_bytree = 0.8  Learning _rate = 0.01  n_estimators = 300  max_depth = 15 | 0.796 | 0.850 | 0.785 | 0.817 | 0.800 | 0.485 | 0.156 |
| SVM | C = 100  Gamma = 1  Kernel = poly | 0.674 | 0.724 | 0.676 | 0.679 | 0.676 | 0.627 | 0.216 |
| MLP | Activation = tanh  Alpha = 0.05  Hidden_layer_sizes = (15,10,5)  Learning_rate = constant  Solver = lbfgs | 0.728 | 0.769 | 0.722 | 0.753 | 0.735 | 1.038 | 0.212 |
| KNN | Metric = euclidean  Weights = distance  N_neighbours = 14 | 0.779 | 0.845 | 0.736 | 0.871 | 0.797 | 0.726 | 0.163 |
| Decision Tree | Max_depth = 16  Criterion = gini  Min_samples_leaf = 5  Min_samples_split = 12 | 0.747 | 0.792 | 0.747 | 0.747 | 0.746 | 3.443 | 0.203 |
| Random Forest | Max_depth = 32  N_estimators = 500  Min_samples_leaf = 3  Min_samples_split = 8 | 0.776 | 0.848 | 0.770 | 0.786 | 0.777 | 0.495 | 0.162 |
| Logistic regression | C = 1  Penalty = l1  Solver = liblinear | 0.674 | 0.708 | 0.662 | 0.716 | 0.687 | 0.628 | 0.219 |
| Naive bayes | Var_smoothing = 0.001873817422860383 | 0.654 | 0.682 | 0.649 | 0.677 | 0.660 | 0.698 | 0.236 |
| Ensemble | XGBoost, Random Forest, Decision tree, KNN | 0.781 | 0.857 | 0.766 | 0.812 | 0.787 | 0.475 | 0.154 |

In case of feature set 4, XGBoost and naive Bayes had the highest (accuracy = 0.796, F1 = 0.800) and poorest performance (accuracy = 0.654, F1 = 0.660), respectively.

Table S10. Performance of “at admission” models on feature set 5

| Algorithm | Parameters | Accuracy | AUC | Precision | Recall | F1 Score | Log Loss | Brier Score |
| --- | --- | --- | --- | --- | --- | --- | --- | --- |
| XGBoost | Colsample_bytree = 0.3  Learning _rate = 0.01  n_estimators = 700  max_depth = 15 | 0.872 | 0.939 | 0.891 | 0.843 | 0.857 | 0.320 | 0.097 |
| SVM | C = 100  Gamma = 1  Kernel = rbf | 0.768 | 0.836 | 0.762 | 0.758 | 0.751 | 0.499 | 0.164 |
| MLP | Activation = relu  Alpha = 0.0001  Hidden_layer_sizes = 15  Learning_rate = constant  Solver = lbfgs | 0.760 | 0.794 | 0.745 | 0.774 | 0.758 | 3.361 | 0.218 |
| KNN | Metric = manhattan  Weights = distance  N_neighbours = 8 | 0.737 | 0.810 | 0.712 | 0.787 | 0.746 | 0.992 | 0.180 |
| Decision Tree | Max_depth = 16  Criterion = entropy  Min_samples_leaf = 3  Min_samples_split = 10 | 0.796 | 0.832 | 0.798 | 0.780 | 0.782 | 4.622 | 0.179 |
| Random Forest | Max_depth = 16  N_estimators = 900  Min_samples_leaf =3  Min_samples_split = 8 | 0.849 | 0.920 | 0.846 | 0.851 | 0.844 | 0.405 | 0.123 |
| Logistic regression | C = 1  Penalty = l1  Solver = liblinear | 0.710 | 0.764 | 0.703 | 0.715 | 0.707 | 0.580 | 0.197 |
| Naive bayes | Var_smoothing = 0.002848035868435802 | 0.676 | 0.721 | 0.673 | 0.676 | 0.673 | 0.683 | 0.224 |
| Ensemble | XGBoost, MLP, Random Forest, Decision Tree | 0.860 | 0.916 | 0.870 | 0.842 | 0.850 | 0.361 | 0.113 |

For feature set 5, XGBoost had the best performance (accuracy = 0.872, F1 = 0.857) while the naive Bayes had the poorest performance (accuracy = 0.676, F1 = 0.673).

Table S11. Performance of “at admission” models on feature set 6

| Algorithm | Parameters | Accuracy | AUC | Precision | Recall | F1 Score | Log Loss | Brier Score |
| --- | --- | --- | --- | --- | --- | --- | --- | --- |
| XGBoost | Colsample_bytree = 0.8  Learning _rate = 0.01  n_estimators = 900  max_depth = 15 | 0.842 | 0.909 | 0.837 | 0.849 | 0.840 | 0.385 | 0.120 |
| SVM | C = 100  Gamma = 1  Kernel = rbf | 0.746 | 0.804 | 0.744 | 0.744 | 0.741 | 0.537 | 0.180 |
| MLP | Activation = logistic  Alpha = 0.05  Hidden_layer_sizes = (15,10)  Learning_rate = constant  Solver = lbfgs | 0.749 | 0.792 | 0.739 | 0.768 | 0.750 | 1.189 | 0.213 |
| KNN | Metric = euclidean  Weights = distance  N_neighbours = 12 | 0.742 | 0.808 | 0.724 | 0.783 | 0.748 | 0.594 | 0.180 |
| Decision Tree | Max_depth = 8  Criterion = entropy  Min_samples_leaf = 3  Min_samples_split = 12 | 0.765 | 0.814 | 0.773 | 0.746 | 0.755 | 2.501 | 0.185 |
| Random Forest | Max_depth = 16  N_estimators = 100  Min_samples_leaf = 3  Min_samples_split = 8 | 0.817 | 0.897 | 0.806 | 0.836 | 0.818 | 0.438 | 0.138 |
| Logistic regression | C = 10  Penalty = l2  Solver = newton-cg | 0.636 | 0.673 | 0.638 | 0.625 | 0.631 | 0.716 | 0.241 |
| Naive bayes | Var_smoothing = 0.001 | 0.676 | 0.721 | 0.673 | 0.676 | 0.673 | 0.683 | 0.224 |
| Ensemble | XGBoost, MLP, Random Forest | 0.815 | 0.903 | 0.813 | 0.822 | 0.812 | 0.406 | 0.130 |

Concerning feature set 6, highest performance is attributed to XGBoost (accuracy = 0.842, F1 = 0.840) while poorest performance is for logistic regression (accuracy = 0.636, F1 = 0.631).

Table S12. Performance of “at admission” models on feature set 7

| Algorithm | Parameters | Accuracy | AUC | Precision | Recall | F1 Score | Log Loss | Brier Score |
| --- | --- | --- | --- | --- | --- | --- | --- | --- |
| XGBoost | Colsample_bytree = 0.3  Learning _rate = 0.01  n_estimators = 500  max_depth = 15 | 0.879 | 0.942 | 0.904 | 0.850 | 0.867 | 0.336 | 0.100 |
| SVM | C = 100  Gamma = 1  Kernel = rbf | 0.788 | 0.859 | 0.787 | 0.776 | 0.777 | 0.463 | 0.151 |
| MLP | Activation = relu  Alpha = 0.05  Hidden_layer_sizes = 10  Learning_rate = constant  Solver = lbfgs | 0.793 | 0.838 | 0.779 | 0.807 | 0.789 | 0.917 | 0.173 |
| KNN | Metric = manhattan  Weights = distance  N_neighbours = 10 | 0.769 | 0.828 | 0.761 | 0.779 | 0.768 | 0.743 | 0.170 |
| Decision Tree | Max_depth = 8  Criterion = gini  Min_samples_leaf = 3  Min_samples_split =12 | 0.808 | 0.855 | 0.805 | 0.802 | 0.797 | 2.222 | 0.158 |
| Random Forest | Max_depth = 32  N_estimators = 900  Min_samples_leaf = 3  Min_samples_split = 8 | 0.852 | 0.925 | 0.854 | 0.844 | 0.844 | 0.397 | 0.121 |
| Logistic regression | C = 1  Penalty = l1  Solver = liblinear | 0.691 | 0.750 | 0.682 | 0.716 | 0.695 | 0.594 | 0.203 |
| Naive bayes | Var_smoothing = 0.0004328761281083057 | 0.654 | 0.691 | 0.636 | 0.740 | 0.682 | 0.889 | 0.255 |
| Ensemble | XGBoost, SVM, Random Forest, Decision Tree, KNN, Logistic Regression | 0.843 | 0.925 | 0.845 | 0.833 | 0.830 | 0.405 | 0.123 |

Regarding feature set 7, best model was XGBoost (accuracy = 0.879, F1 = 0.867) and poorest model was naive Bayes (accuracy = 0.654, F1 = 0.682).

Table S13. Performance of “at admission” models on feature set 8

| Algorithm | Parameters | Accuracy | AUC | Precision | Recall | F1 Score | Log Loss | Brier Score |
| --- | --- | --- | --- | --- | --- | --- | --- | --- |
| XGBoost | Colsample_bytree = 0.5  Learning _rate = 0.01  n_estimators = 300  max_depth = 15 | 0.867 | 0.929 | 0.860 | 0.872 | 0.864 | 0.366 | 0.109 |
| SVM | C = 0.1  Gamma = 1  Kernel = poly | 0.776 | 0.826 | 0.743 | 0.839 | 0.785 | 0.536 | 0.171 |
| MLP | Activation = relu  Alpha = 0.0001  Hidden_layer_sizes = (15,10)  Learning_rate = constant  Solver = lbfgs | 0.775 | 0.807 | 0.771 | 0.778 | 0.771 | 3.844 | 0.208 |
| KNN | Metric = euclidean  Weights = distance  N_neighbours = 6 | 0.747 | 0.817 | 0.709 | 0.820 | 0.758 | 1.518 | 0.181 |
| Decision Tree | Max_depth = 16  Criterion = entropy  Min_samples_leaf = 3  Min_samples_split = 8 | 0.778 | 0.810 | 0.786 | 0.757 | 0.765 | 5.105 | 0.195 |
| Random Forest | Max_depth = 32  N_estimators = 700  Min_samples_leaf = 3  Min_samples_split = 8 | 0.842 | 0.913 | 0.828 | 0.852 | 0.838 | 0.413 | 0.127 |
| Logistic regression | C = 1  Penalty = l1  Solver = liblinear | 0.734 | 0.792 | 0.715 | 0.765 | 0.738 | 0.555 | 0.185 |
| Naive bayes | Var_smoothing = 0.12328467394420659 | 0.672 | 0.758 | 0.618 | 0.892 | 0.729 | 0.864 | 0.251 |
| Ensemble | XGBoost, MLP, Random Forest, Decision tree, KNN | 0.837 | 0.915 | 0.825 | 0.846 | 0.832 | 0.377 | 0.118 |

For feature set 8, best model was the XGBoost (accuracy = 0.867, F1 = 0.864) and poorest one was the naive Bayes (accuracy = 0.672, F1 = 0.729).

Table S14. Performance of “post admission” models on feature set 1

| Algorithm | Parameters | Accuracy | AUC | Precision | Recall | F1 Score | Log Loss | Brier Score |
| --- | --- | --- | --- | --- | --- | --- | --- | --- |
| XGBoost | Colsample_bytree = 0.3  Learning _rate = 0.01  n_estimators = 900  max_depth = 10 | 0.897 | 0.945 | 0.915 | 0.868 | 0.886 | 0.257 | 0.078 |
| SVM | C = 100  Gamma = 0.1  Kernel = rbf | 0.824 | 0.880 | 0.831 | 0.807 | 0.816 | 0.428 | 0.131 |
| MLP | Activation = logistic  Alpha = 0.05  Hidden_layer_sizes = 15  Learning_rate = constant  Solver = lbfgs | 0.820 | 0.870 | 0.812 | 0.828 | 0.817 | 0.704 | 0.151 |
| KNN | Metric = manhattan  Weights = distance  N_neighbours = 10 | 0.820 | 0.866 | 0.817 | 0.831 | 0.823 | 1.111 | 0.143 |
| Decision Tree | Max_depth = 16  Criterion = gini  Min_samples_leaf = 3  Min_samples_split = 12 | 0.857 | 0.888 | 0.861 | 0.853 | 0.854 | 2.835 | 0.124 |
| Random Forest | Max_depth = 16  N_estimators = 300  Min_samples_leaf = 3  Min_samples_split = 10 | 0.886 | 0.926 | 0.900 | 0.866 | 0.880 | 0.320 | 0.095 |
| Logistic regression | C = 1000  Penalty = l1  Solver = liblinear | 0.791 | 0.857 | 0.801 | 0.785 | 0.791 | 0.487 | 0.153 |
| Naive bayes | Var_smoothing = 0.3511191734215131 | 0.724 | 0.829 | 0.670 | 0.899 | 0.767 | 0.576 | 0.188 |
| Ensemble | XGBoost, SVM, MLP, Random Forest, Decision Tree | 0.877 | 0.929 | 0.882 | 0.868 | 0.872 | 0.306 | 0.092 |

In feature set 1, the best and poorest performance was for XGBoost (accuracy = 0.897, F1 = 0.886), and naive bayes (accuracy = 0.724, F1 = 0.767).

Table S15. Performance of “post admission” models on feature set 2

| Algorithm | Parameters | Accuracy | AUC | Precision | Recall | F1 Score | Log Loss | Brier Score |
| --- | --- | --- | --- | --- | --- | --- | --- | --- |
| XGBoost | Colsample_bytree =0.3  Learning _rate = 0.01  n_estimators = 900  max_depth = 15 | 0.901 | 0.947 | 0.931 | 0.861 | 0.885 | 0.251 | 0.075 |
| SVM | C = 100  Gamma = 0.01  Kernel = rbf | 0.820 | 0.875 | 0.825 | 0.818 | 0.821 | 0.447 | 0.138 |
| MLP | Activation = tanh  Alpha = 0.0001  Hidden_layer_sizes = 10  Learning_rate = constant  Solver = adam | 0.811 | 0.887 | 0.800 | 0.831 | 0.813 | 0.492 | 0.138 |
| KNN | Metric = manhattan  Weights = distance  N_neighbours = 16 | 0.808 | 0.873 | 0.782 | 0.866 | 0.821 | 0.437 | 0.141 |
| Decision Tree | Max_depth = 16  Criterion = gini  Min_samples_leaf = 3  Min_samples_split = 12 | 0.849 | 0.880 | 0.855 | 0.844 | 0.845 | 2.823 | 0.130 |
| Random Forest | Max_depth = 32  N_estimators = 300  Min_samples_leaf = 3  Min_samples_split = 10 | 0.885 | 0.928 | 0.896 | 0.870 | 0.880 | 0.315 | 0.093 |
| Logistic regression | C = 10  Penalty = l2  Solver = newton-cg | 0.812 | 0.863 | 0.810 | 0.820 | 0.814 | 0.467 | 0.147 |
| Naive bayes | Var_smoothing = 2.848035868435799e-07 | 0.716 | 0.843 | 0.656 | 0.921 | 0.766 | 1.661 | 0.237 |
| Ensemble | XGBoost, SVM, Random Forest, Decision Tree, KNN | 0.885 | 0.928 | 0.890 | 0.877 | 0.881 | 0.317 | 0.093 |

For feature set 2, XGBoost showed the best performance (accuracy = 0.901, F1 = 0.855); however, naive Bayes had the poorest performance (accuracy = 0.716, F1 = 0.766).

Table S16. Performance of “post admission” models on feature set 3

| Algorithm | Parameters | Accuracy | AUC | Precision | Recall | F1 Score | Log Loss | Brier Score |
| --- | --- | --- | --- | --- | --- | --- | --- | --- |
| XGBoost | Colsample_bytree = 0.5  Learning _rate = 0.1  n_estimators = 300  max_depth = 8 | 0.901 | 0.945 | 0.904 | 0.897 | 0.897 | 0.282 | 0.080 |
| SVM | C = 100  Gamma = 0.1  Kernel = rbf | 0.803 | 0.868 | 0.811 | 0.789 | 0.798 | 0.461 | 0.140 |
| MLP | Activation = logistic  Alpha = 0.0001  Hidden_layer_sizes = 5  Learning_rate = constant  Solver = lbfgs | 0.811 | 0.861 | 0.830 | 0.787 | 0.805 | 1.088 | 0.157 |
| KNN | Metric = manhattan  Weights = distance  N_neighbours = 20 | 0.822 | 0.866 | 0.816 | 0.842 | 0.828 | 0.535 | 0.143 |
| Decision Tree | Max_depth = 16  Criterion = entropy  Min_samples_leaf = 3  Min_samples_split = 12 | 0.843 | 0.872 | 0.847 | 0.833 | 0.837 | 3.541 | 0.137 |
| Random Forest | Max_depth = 16  N_estimators = 100  Min_samples_leaf = 3  Min_samples_split = 8 | 0.881 | 0.932 | 0.891 | 0.870 | 0.878 | 0.321 | 0.094 |
| Logistic regression | C = 100  Penalty = l1  Solver = liblinear | 0.781 | 0.838 | 0.778 | 0.794 | 0.785 | 0.502 | 0.162 |
| Naive bayes | Var_smoothing = 0.003511191734215131 | 0.746 | 0.802 | 0.744 | 0.769 | 0.754 | 0.597 | 0.188 |
| Ensemble | XGBoost, SVM, MLP, Random Forest, Decision Tree, KNN | 0.885 | 0.926 | 0.897 | 0.872 | 0.881 | 0.324 | 0.096 |

In case of feature set 3, XGBoost outperformed all the other models (accuracy = 0.901, F1 = 0.897) while naive Bayes was inferior (accuracy = 0.746, F1 = 0.754).

Table S17. Performance of “post admission” models on feature set 4

| Algorithm | Parameters | Accuracy | AUC | Precision | Recall | F1 Score | Log Loss | Brier Score |
| --- | --- | --- | --- | --- | --- | --- | --- | --- |
| XGBoost | Colsample_bytree = 0.8  Learning _rate = 0.01  n_estimators = 900  max_depth = 4 | 0.830 | 0.902 | 0.855 | 0.805 | 0.826 | 0.382 | 0.123 |
| SVM | C = 100  Gamma = 1  Kernel = rbf | 0.803 | 0.874 | 0.817 | 0.801 | 0.806 | 0.491 | 0.147 |
| MLP | Activation = tanh  Alpha = 0.0001  Hidden_layer_sizes = (15,10)  Learning_rate = constant  Solver = lbfgs | 0.822 | 0.878 | 0.856 | 0.772 | 0.810 | 0.522 | 0.134 |
| KNN | Metric = euclidean  Weights = uniform  N_neighbours = 20 | 0.807 | 0.889 | 0.849 | 0.753 | 0.796 | 0.513 | 0.132 |
| Decision Tree | Max_depth = 4  Criterion = entropy  Min_samples_leaf = 5  Min_samples_split = 8 | 0.833 | 0.895 | 0.869 | 0.785 | 0.822 | 0.580 | 0.123 |
| Random Forest | Max_depth = 8  N_estimators = 100  Min_samples_leaf = 3  Min_samples_split = 12 | 0.828 | 0.898 | 0.862 | 0.790 | 0.821 | 0.381 | 0.124 |
| Logistic regression | C = 1  Penalty = l2  Solver = newton-cg | 0.750 | 0.828 | 0.787 | 0.712 | 0.743 | 0.547 | 0.181 |
| Naive bayes | Var_smoothing = 0.533669923120631 | 0.732 | 0.830 | 0.784 | 0.671 | 0.717 | 0.582 | 0.196 |
| Ensemble | XGBoost, SVM, Random Forest, Logistic Regression, Naïve Bayes | 0.809 | 0.893 | 0.815 | 0.818 | 0.815 | 0.437 | 0.136 |

As for feature set 4, XGBoost and naive Bayes had the best (accuracy = 0.830, F1 = 0.826) and lowest performance (accuracy = 0.732, F1 = 0.717), respectively.

Table S18. Performance of “post admission” models on feature set 5

| Algorithm | Parameters | Accuracy | AUC | Precision | Recall | F1 Score | Log Loss | Brier Score |
| --- | --- | --- | --- | --- | --- | --- | --- | --- |
| XGBoost | Colsample_bytree = 0.3  Learning _rate = 0.1  n_estimators = 300  max_depth = 15 | 0.904 | 0.946 | 0.915 | 0.892 | 0.899 | 0.268 | 0.076 |
| SVM | C = 100  Gamma = 0.1  Kernel = rbf | 0.799 | 0.856 | 0.795 | 0.803 | 0.798 | 0.479 | 0.146 |
| MLP | Activation = tanh  Alpha = 0.05  Hidden_layer_sizes = 5  Learning_rate = constant  Solver = lbfgs | 0.806 | 0.869 | 0.788 | 0.833 | 0.808 | 0.608 | 0.148 |
| KNN | Metric = manhattan  Weights = distance  N_neighbours = 20 | 0.812 | 0.859 | 0.810 | 0.823 | 0.816 | 0.570 | 0.147 |
| Decision Tree | Max_depth = 16  Criterion = entropy  Min_samples_leaf = 3  Min_samples_split = 12 | 0.850 | 0.875 | 0.853 | 0.842 | 0.844 | 3.442 | 0.133 |
| Random Forest | Max_depth = 16  N_estimators = 100  Min_samples_leaf = 3  Min_samples_split = 8 | 0.887 | 0.934 | 0.899 | 0.875 | 0.884 | 0.321 | 0.094 |
| Logistic regression | C = 100  Penalty = l1  Solver = liblinear | 0.778 | 0.842 | 0.779 | 0.785 | 0.780 | 0.497 | 0.160 |
| Naive bayes | Var_smoothing = 0.004328761281083057 | 0.749 | 0.805 | 0.753 | 0.762 | 0.755 | 0.581 | 0.184 |
| Ensemble | XGBoost, SVM, Random Forest, Decision Tree, Logisitc Regression | 0.881 | 0.926 | 0.890 | 0.872 | 0.877 | 0.327 | 0.095 |

For feature set 5, XGBoost had the best performance (accuracy = 0.904, F1 = 0.899) and naive Bayes was attributed with weakest performance (accuracy = 0.749, F1 = 0.755).

Table S19. Performance of “post admission” models on feature set 6

| Algorithm | Parameters | Accuracy | AUC | Precision | Recall | F1 Score | Log Loss | Brier Score |
| --- | --- | --- | --- | --- | --- | --- | --- | --- |
| XGBoost | Colsample_bytree = 0.3  Learning _rate = 0.01  n_estimators = 700  max_depth =8 | 0.896 | 0.937 | 0.924 | 0.857 | 0.884 | 0.292 | 0.085 |
| SVM | C = 100  Gamma = 1  Kernel = rbf | 0.838 | 0.897 | 0.861 | 0.800 | 0.828 | 0.382 | 0.121 |
| MLP | Activation = logistic  Alpha = 0.05  Hidden_layer_sizes = (15,10)  Learning_rate = constant  Solver = lbfgs | 0.849 | 0.891 | 0.849 | 0.844 | 0.844 | 0.943 | 0.133 |
| KNN | Metric = manhattan  Weights = distance  N_neighbours = 6 | 0.820 | 0.878 | 0.830 | 0.809 | 0.818 | 1.455 | 0.136 |
| Decision Tree | Max_depth = 4  Criterion = gini  Min_samples_leaf = 3  Min_samples_split = 8 | 0.831 | 0.862 | 0.817 | 0.851 | 0.833 | 0.669 | 0.138 |
| Random Forest | Max_depth = 16  N_estimators = 300  Min_samples_leaf = 3  Min_samples_split = 8 | 0.881 | 0.925 | 0.884 | 0.877 | 0.878 | 0.319 | 0.094 |
| Logistic regression | C = 1000  Penalty = l2  Solver =lbfgs | 0.771 | 0.850 | 0.779 | 0.768 | 0.772 | 0.495 | 0.160 |
| Naive bayes | Var_smoothing = 0.8111308307896871 | 0.761 | 0.829 | 0.744 | 0.818 | 0.778 | 0.560 | 0.187 |
| Ensemble | XGBoost, MLP, Random Forest | 0.890 | 0.936 | 0.892 | 0.883 | 0.886 | 0.291 | 0.086 |

Concerning feature set 6, ensemble model showed the highest performance (accuracy = 0.890, F1 = 0.886) and naive Bayes showed lowest performance (accuracy = 0.761, F1 = 0.778).

Table S20. Performance of “post admission” models on feature set 7

| Algorithm | Parameters | Accuracy | AUC | Precision | Recall | F1 Score | Log Loss | Brier Score |
| --- | --- | --- | --- | --- | --- | --- | --- | --- |
| XGBoost | Colsample_bytree = 0.5  Learning _rate = 0.01  n_estimators = 500  max_depth = 10 | 0.902 | 0.950 | 0.919 | 0.883 | 0.896 | 0.255 | 0.075 |
| SVM | C = 100  Gamma = 1  Kernel = rbf | 0.841 | 0.894 | 0.845 | 0.831 | 0.836 | 0.389 | 0.121 |
| MLP | Activation = logistic  Alpha = 0.05  Hidden_layer_sizes = 10  Learning_rate = constant  Solver = lbfgs | 0.828 | 0.884 | 0.830 | 0.822 | 0.823 | 0.602 | 0.137 |
| KNN | Metric = manhattan  Weights = distance  N_neighbours = 12 | 0.842 | 0.896 | 0.845 | 0.853 | 0.846 | 0.674 | 0.123 |
| Decision Tree | Max_depth = 16  Criterion = gini  Min_samples_leaf = 5  Min_samples_split = 8 | 0.836 | 0.873 | 0.833 | 0.835 | 0.831 | 2.837 | 0.135 |
| Random Forest | Max_depth = 16  N_estimators = 500  Min_samples_leaf = 3  Min_samples_split = 8 | 0.882 | 0.935 | 0.888 | 0.877 | 0.880 | 0.309 | 0.091 |
| Logistic regression | C = 1  Penalty = l1  Solver = liblinear | 0.791 | 0.855 | 0.803 | 0.783 | 0.791 | 0.485 | 0.156 |
| Naive bayes | Var_smoothing = 0.7178389398572886 | 0.718 | 0.821 | 0.684 | 0.832 | 0.749 | 1.156 | 0.212 |
| Ensemble | XGBoost, SVM, Random Forest, Decision Tree | 0.881 | 0.934 | 0.891 | 0.868 | 0.876 | 0.297 | 0.088 |

Regarding feature set 7, the best and weakest performance is attributed to XGBoost (accuracy = 0.902, F1 = 0.896), and naive Bayes (accuracy = 0.718, F1 = 0.749).

Table S21. Performance of “post admission” models on feature set 8

| Algorithm | Parameters | Accuracy | AUC | Precision | Recall | F1 Score | Log Loss | Brier Score |
| --- | --- | --- | --- | --- | --- | --- | --- | --- |
| XGBoost | Colsample_bytree = 0.3  Learning _rate = 0.01  n_estimators = 300  max_depth = 15 | 0.909 | 0.952 | 0.921 | 0.894 | 0.904 | 0.323 | 0.090 |
| SVM | C = 100  Gamma = 1  Kernel = poly | 0.816 | 0.857 | 0.804 | 0.833 | 0.816 | 0.501 | 0.155 |
| MLP | Activation = tanh  Alpha = 0.05  Hidden_layer_sizes = 10  Learning_rate = constant  Solver = lbfgs | 0.826 | 0.869 | 0.816 | 0.839 | 0.824 | 1.310 | 0.157 |
| KNN | Metric = manhattan  Weights = distance  N_neighbours = 6 | 0.817 | 0.871 | 0.797 | 0.860 | 0.826 | 1.405 | 0.139 |
| Decision Tree | Max_depth = 16  Criterion = gini  Min_samples_leaf = 3  Min_samples_split = 10 | 0.857 | 0.888 | 0.853 | 0.864 | 0.856 | 2.945 | 0.123 |
| Random Forest | Max_depth = 16  N_estimators = 300  Min_samples_leaf = 3  Min_samples_split = 8 | 0.884 | 0.932 | 0.893 | 0.872 | 0.880 | 0.323 | 0.094 |
| Logistic regression | C = 10  Penalty = l2  Solver =newton-cg | 0.794 | 0.860 | 0.795 | 0.801 | 0.797 | 0.471 | 0.150 |
| Naive bayes | Var_smoothing = 2.310129700083158e-08 | 0.733 | 0.832 | 0.687 | 0.877 | 0.769 | 1.503 | 0.219 |
| Ensemble | XGBoost, SVM, Random Forest, Decision Tree, KNN | 0.891 | 0.936 | 0.885 | 0.899 | 0.890 | 0.326 | 0.094 |

For feature set 8, the highest performance was for XGBoost (accuracy = 0.909, F1 = 0.904) and lowest performance was attributed to naive Bayes (accuracy = 0.733, F1 = 0.769).
