# Supplementary material for: Machine learning-based mortality prediction models for smoker COVID-19 patients
Source: BMC Med Inform Decis Mak. 2023 Jul 21;23:129. doi: 10.1186/s12911-023-02237-w (PMC10360290; doi:10.1186/s12911-023-02237-w)
Supplement: Supplementary file 2 — Supplementary Material 2 [file 12911_2023_2237_MOESM2_ESM.docx]

Supplementary Data (Figures)

Content

[Figure S1. Top 20 important features for at admission models based on extratreesclassifier Gini importance 2](#_Toc128489177)

[Figure S2. Top 20 important features for at admission models based on random forest Gini importance 3](#_Toc128489178)

[Figure S3. Top 20 important features for at admission models based on gradient boosting Gini importance 4](#_Toc128489179)

[Figure S4. Top 20 important features for post admission models based on extratreesclassifier Gini importance 5](#_Toc128489180)

[Figure S5. Top 20 important features for post admission models based on random forest Gini importance 6](#_Toc128489181)

[Figure S6. Top 20 important features for post admission models based on gradient boosting Gini importance 7](#_Toc128489182)

[Figure S7. Current smoking SHAP dependence plots for "at admission" model 8](#_Toc128489183)

[Figure S8. Current smoking SHAP dependence plots for “post admission” model 9](#_Toc128489184)


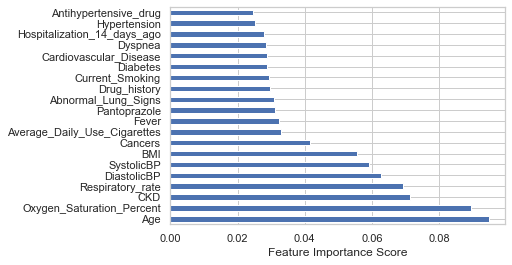


Figure S1. Top 20 important features for at admission models based on extratreesclassifier Gini importance


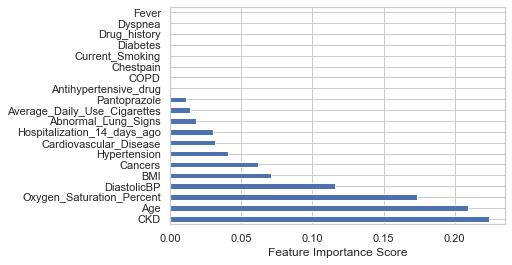


Figure S2. Top 20 important features for at admission models based on random forest Gini importance


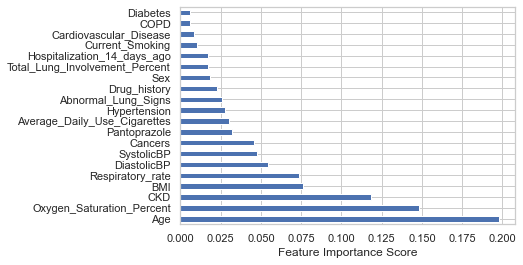


Figure S3. Top 20 important features for at admission models based on gradient boosting Gini importance


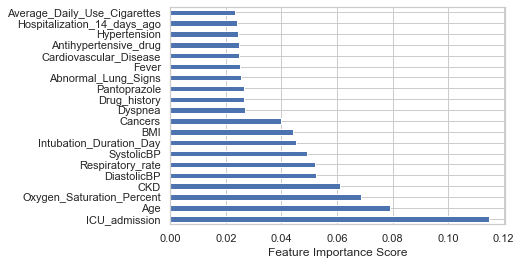


Figure S4. Top 20 important features for post admission models based on extratreesclassifier Gini importance


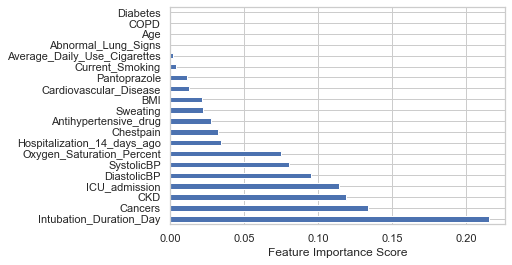


Figure S5. Top 20 important features for post admission models based on random forest Gini importance


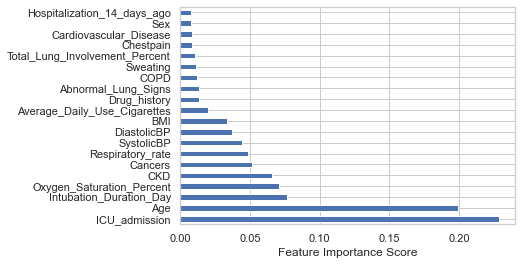


Figure S6. Top 20 important features for post admission models based on gradient boosting Gini importance


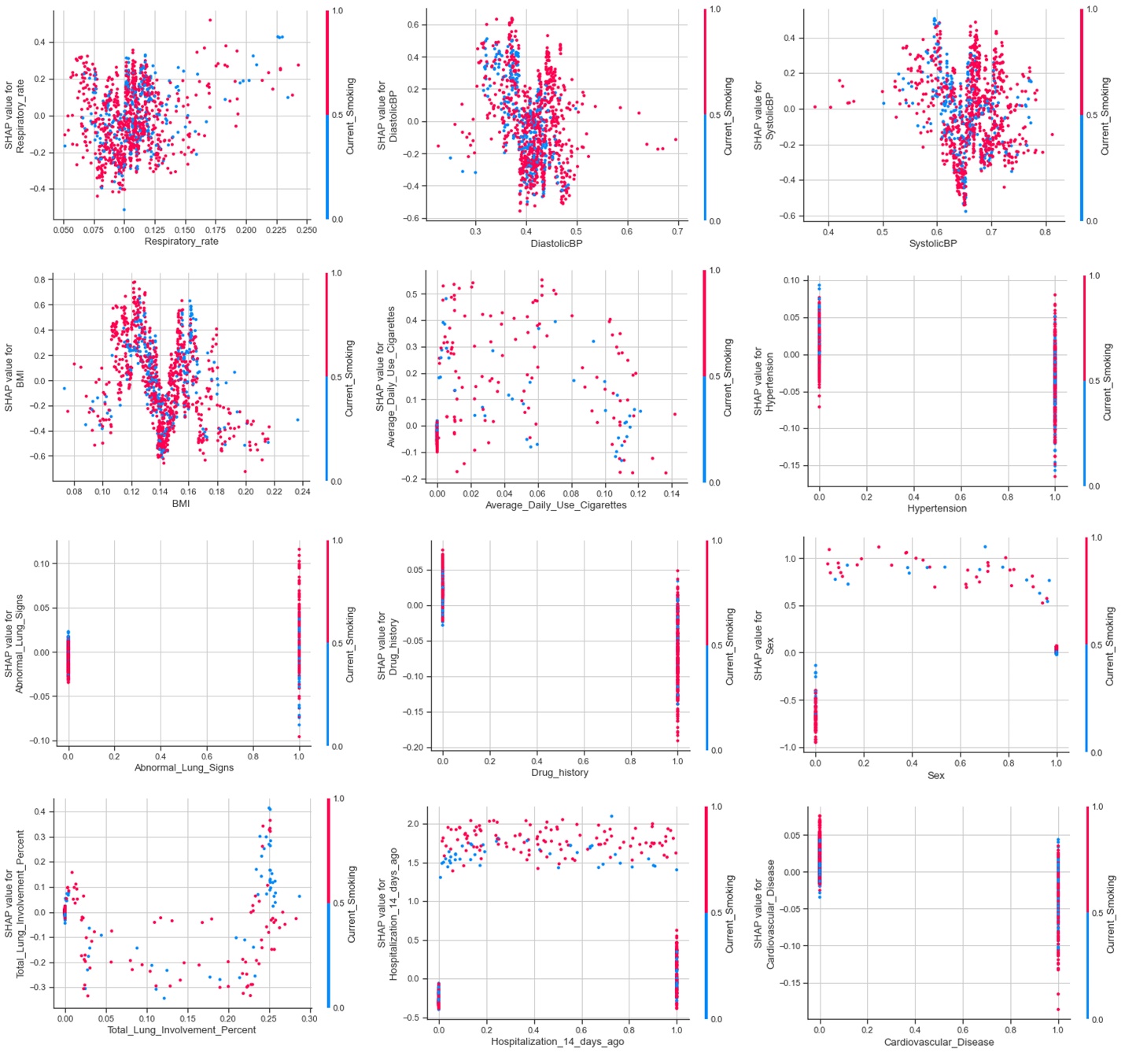


Figure S7. Current smoking SHAP dependence plots for “at admission” model


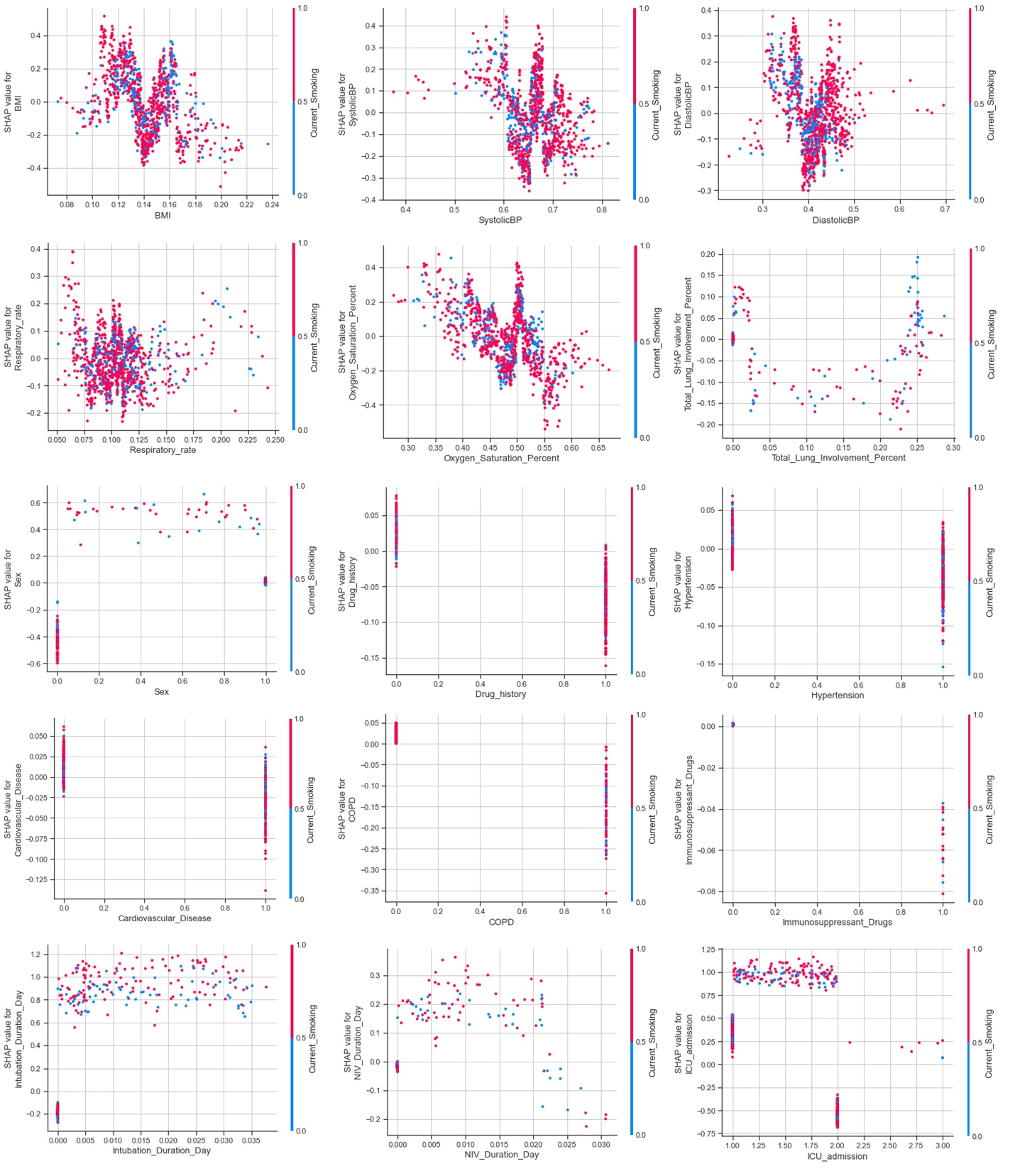


Figure S8. Current smoking SHAP dependence plots for “post admission” model
